# Adaptive aggregation for longitudinal quantile regression based on censored history process

**DOI:** 10.1177/09622802231164730

**Published:** 2023-03-28

**Authors:** Wei Xiong, Dianliang Deng, Dehui Wang, Wanying Zhang

**Affiliations:** 1 School of Mathematics, 12510 Jilin University, Changchun, China; 2 Department of Mathematics and Statistics, 6846 University of Regina, Regina, SK, Canada; 3 School of Mathematics and Statistics, 12440 Liaoning University, Shenyang, China

**Keywords:** Aggregation, longitudinal quantile regression, random effect, right censoring, smoothing loss function

## Abstract

Most of the studies for longitudinal quantile regression are based on the correct specification. Nevertheless, one specific model can hardly perform precisely under different conditions and assessing which conditions are (approximately) satisfied to determine the optimal one is rather difficult. In the case of the mixed effect model, the misspecification of the fixed effect part will cause a lack of predicting accuracy of random effects, and affect the efficiency of the cumulative function estimator. On the other hand, limited research has focused on incorporating multiple candidate procedures in longitudinal data analysis, which is of current emergency. This paper proposes an exponential aggregation weighting algorithm for longitudinal quantile regression. Based on the secondary smoothing loss function, we establish oracle inequalities for aggregated estimator. The proposed method is applied to evaluate the cumulative τ th quantile function for additive mixed effect model with right-censored history process, and an aggregation-based best linear prediction for random effects is constructed as well. We show that the asymptotic properties are conveniently imposed owing to the smoothing scheme. Simulation studies are carried out to exhibit the rationality, and our method is illustrated to analyze the data set from a multicenter automatic defibrillator implantation trial.

## Introduction

Quantile regression (Koenker and Bassett^1^) is an increasingly prevalent tool. It provides a constructive strategy for examining the effect of covariates on the entire response distribution and offers a more flexible, robust approach for data analysis. In longitudinal studies, the conditional τ th quantile regression of a given probability level τ∈(0,1) is assumed as

(1) $$Q_{\tau }(y(t)|x(t))=q_{\tau }(x(t))$$

where y(t) , x(t) is the response variable and p×1 dimensional covariate vector at time t , respectively. $q_{\tau }(x(t))$ is an unspecified function which contains conditional τ th quantile of model errors among x(t) . For a determined $q_{\tau }(x(t))$ , there is a great deal of literature for related statistical inferences. Yin and Cai^2^ proposed a working independent method for linear quantile regression (LQR) with correlated failure time data. Lu and Fan^3^ estimated parameters in weighted quantile regression. Tang et al.^4^ proposed a B-spline-based variable selection procedure for varying-coefficient quantile regression, and Wang and Sun^5^ extended the approach to partial linear varying coefficient model with the within-subject correlation structure.

One of the significances for longitudinal quantile regression is dealing with censoring. Galvao et al.^6^ proposed a three-step estimator in the sense of panel quantile regression with fixed individual effects and left censoring. Harding and Lamarche^7^ developed a penalized estimation with a parametric or nonparametric mechanism to reduce attrition bias in Big Data, which is applicable to censored data as well. Recently, Liu et al.^8^ discussed the estimation of the cumulative τ th quantile function ( τ -CQF) for history process with right-censored time-to-event variable. Nevertheless, most of the existing methodologies are based on the fixed specification of model (1). Actually, there are a number of sampling designs, which require consideration for the correlation between observations that belongs to the same unit or cluster. The random effect provides a flexible medium in terms of complex data analysis. Koenker,^9^ Geraci and Bottai,^10^ and Kulkarni et al.^11^ generalized equation (1) to the longitudinal quantile mixed effect model (LQMM) with linear fixed effect and random intercepts. More recently, Geraci^12^ developed the algorithm for the covariate-affected random effect structure by Gaussian quadrature technique, and Geraci^13^ proposed a spline-based approximation for the nonparametric additive form of the fixed effect pattern to make an inference.

In practice, identifying an extremely correct model is rather difficult for the data at hand. Traditional model selection is commonly used to search for the most approximate one. Machado^14^ proposed a robust selection criterion for general M-estimators which is applicable to quantile regression, and Koenker^15^ suggested the traditional Akaike information criterion. The shortcoming of the above methods is frequently ignoring the level of uncertainty associated with the choice of the model while reporting precision estimates. One approach to incorporating such uncertainty is model averaging, which combines competing specifications via propensity weights. Since the pioneering contribution of Hjort and Claeskens,^16^ the number of works on the topic has proliferated in the past few years. For instance, Hansen^17^ proposed the Mallows’ criterion for selecting weights of least squares regressive models; Lu and Su^18^ used a jackknife model averaging strategy to sort weights in general quantile regression; and Wang et al.^19^ generalized the method with high-dimensional covariates, etc.

It is noted that the aforementioned thoughts are based on the weights via vested information criterion, and emphasize the parametric model assumption. To make the comparison with complex nonparametric forms, Yang^20^ proposed an adaptive estimation for least squares regression by model mixing. Recently, Shan and Yang,^21^ Gu and Zou^22^ imposed the method to contribute the aggregation algorithm for quantile regression and expectile regression, respectively. The estimators are theoretically shown to approach the best candidate. However, few literary works considered the aggregation for longitudinal quantile regression, especially in view of τ -CQF evaluating. To address the void, we contribute an adaptive estimation for longitudinal quantile regression. An exponential aggregation weighting (EAW) algorithm is adopted to combine multiple candidate estimating procedures, which efficiently guards against the misspecification of the fixed effect $q_{\tau }(x(t))$ and exhibits optimally without knowing which of the original procedures works the best.

In an effort to acquire the asymptotic properties of aggregated estimator, we establish the oracle inequalities under particular risk measures. Since the risk bound under the squared error loss can be hardly derived in quantile regression, (Gu and Zou^22^), we propose a novel smoothing scheme for the quantile check loss function, which supplies the strong convexity and the L -smoothness. Furthermore, we utilize EAW algorithm to additive mixed effect model and construct the estimation of τ -CQF. One can inspect that the estimator behaves consistently with that of the best procedure under the secondary smoothing loss. We also develop an EAW-based best linear prediction (EAW-BLP) of random effects via the proposed weights. A real dataset from multicenter automatic defibrillator implantation trial (MADIT) is analyzed to report the usability of the proposed methodology.

The rest of the paper is organized as follows. In Section 2.1 we introduce the EAW algorithm for (1). We propose a secondary smoothing approximation to check the loss function and employ it during the weighting algorithm. We apply EAW algorithm to additive mixed effect model and propose an estimator for τ -CQF based on right-censored history process in Section 2.2, and the prediction of the random effects is modified with the aggregated weights in Section 2.3. We establish oracle inequalities of EAW estimator under original prediction risk and squared loss in Section 3.1, and related consistency of τ -CQF estimator in Section 3.2, respectively. Simulation studies are presented in Section 4.1 to examine the validity of our algorithm, and we check the predicting accuracy of EAW-BLP for random effects in Section 4.2. We use the method to the MADIT dataset in Section 5. All technical proofs are attached in Appendix.

## 1. Methodology

In this section, we establish several aggregated estimators for longitudinal quantile regression. We firstly propose the EAW algorithm in the sense of model (1), and apply it to evaluate the cumulative τ th quantile function ( τ -CQF) for additive mixed effect model with right-censored history process. In addition, the prediction of the random effects is discussed in the aggregation framework as well.

### 2.1 EAW algorithm

Suppose there is a sample of n subjects to be observed and for the i th subject, $y_{i}(t_{ij})$ and $x_{i}(t_{ij})$ are collected at time points $t_{ij}$ ( $j=1,\ldots ,n_{i}$ ), where $n_{i}$ is the respective total number of observations. Assume that the observed times of all subjects are bounded by T . There are a large corpus of strategies on obtaining a consistent estimator from observation pairs $\left(x_{i}(t_{ij}),y_{i}(t_{ij}),t_{ij}\right)$ if the structure of equation (1) is known. However, the specification may be changeable in different contexts and a unique estimator is sensitive to the failure of efficiency. Let $\Delta =\{\delta_{k}:k>1\}$ be a set of candidate procedures and $\delta_{k}$ be the k th procedure of $q_{\tau }(x(t))$ . Further, let ${\hat{q}}_{\tau }^{\left(k\right)}(x(t))$ be the related estimator from $\delta_{k}$ . There is no special restriction on $\delta_{k}$ as they can be either parametric or nonparametric. For instance, $\delta_{1}$ is a standard parametric linear regression, $\delta_{2}$ is a varying-coefficient regression, $\delta_{3}$ represents the nonparametric additive model, etc. On the other hand, the cardinality of Δ can be either finite or countably infinite. The goal in this section is to combine multiple candidate procedures in Δ to produce an adaptive estimator.

Gu and Zou^22^ proposed the aggregated regression for the asymmetric squared loss. Note that for quantile regression the loss function is $\rho_{\tau }(v)=v(\tau -I(v<0))$ .^15^ One can hardly obtain a similar inequality with (2.4) in Gu and Zou^22^ since it cannot be justified as strongly convex or L -smooth, while this will provide a succinct course in the follow-up theoretical study. Although Shan and Yang^21^ considered a surrogate of $\rho_{\tau }(v)$ , which is nondifferentiable at v=0 , they only contributed the oracle inequality via the surrogate loss. Comparatively, directly smoothing strategies such as piecewise of $\rho_{\tau }(v)$ ^23,24^ involve the first order power (or equivalently, the absolute) of v , and thus it is hard to guarantee the strong convexity, which is essential to attain the risk bound under the squared error loss. To overcome the difficulty, we propose a secondary smoothing approximation of $\rho_{\tau }(v)$ that

(2) $$\rho_{\tau ,h}^{*}(v)=\rho_{\tau ,h}(v)+a(h)v^{2}$$

where a(h) is a function of h on (0,+∞) , and $\rho_{\tau ,h}(v)$ is the smoothing approximation of $\rho_{\tau }(v)$ that is convex and L -smooth in R . In this paper we use the structure of Aravkin et al.^24^ as

$$\rho_{\tau ,h}(v)=\left\{ \begin{array}{l} (1-\tau )|v|-h(1-\tau {)}^{2}/2,\;\;v<-(1-\tau )h,\\ v^{2}/(2h),\;\;\;\;-(1-\tau )h\leq v\leq \tau h,\\ \tau |v|-h\tau^{2}/2,\;\;\;\;v>\tau h. \end{array}\right.$$

From Lemma 1 in Appendix, it can be proved that $\rho_{\tau ,h}^{*}(v)$ is strongly convex, L -smooth and has the minimum at the same point as $\rho_{\tau }(v)$ .

**Figure 1.**
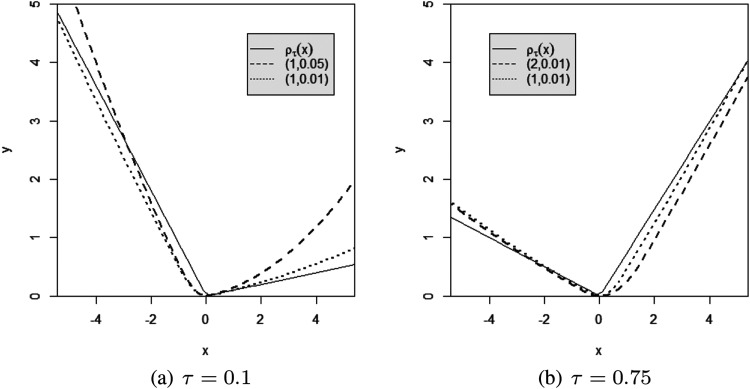
The curves comparing with the proposed secondary smoothing loss function and the original check loss function. Different dotted lines represent the secondary smoothing loss under different values of (h,a(h)) , and the solid line is the original check loss.

There are two smoothing coefficients in the secondary smoothing loss function (2). Figure 1 plots the curves of $\rho_{\tau ,h}^{*}(v)$ under varying pairs of (h,a(h)) . One can see that the skewness between the tails of $\rho_{\tau ,h}^{*}(v)$ and $\rho_{\tau }(v)$ is dominated by a(h) and the global approximation for original check loss is adjudicated by h . Therefore, in order to warrant $\rho_{\tau ,h}^{*}(v)$ close to $\rho_{\tau }(v)$ across the board, a(h)h should be adjusted and a(h) be evaluated smaller than h . Related discussions of the constraints are presented in the next sections to protect the asymptotic efficiency for the aggregated estimation.

In what follows, we propose EAW algorithm for longitudinal quantile regression. The algorithm is based on the multiple splits for the observed dataset, which is typically considered in machine learning, and utilizes the secondary smoothing loss during the weighting process.

**Algorithm 1**

Step 1. ( b th Random Split) Given 1≤b≤B , let $\left(x_{i}^{b},y_{i}^{b},t_{i}^{b}\right)$ denote the b th randomly permuted data of $\left(x_{i},y_{i},t_{i}\right)$ via subject size (where $x_{i}=(x_{i}(t_{i1}),\ldots ,x_{i}(t_{in_{i}}){)}^{⊤}$ , $y_{i}=(y_{i}(t_{i1}),\ldots ,y_{i}(t_{in_{i}}){)}^{⊤}$ , $t_{i}=(t_{i1},\ldots ,t_{in_{i}}{)}^{⊤}$ ), and $N_{0}=\max(1,\lfloor cn\rfloor )$ for some 0<c<1 . Split the sort into a training set $N_{1}^{b}=\{x_{i}^{b},y_{i}^{b},t_{i}^{b}{\}}_{i=1}^{N_{0}}$ and a test set $N_{2}^{b}=\{x_{i}^{b},y_{i}^{b},t_{i}^{b}{\}}_{i=N_{0}+1}^{n}$ .
Step 2. For each procedure $\delta_{k}\in \Delta$ , estimate the conditional quantile in equation (1) by $N_{1}^{b}$ , denoted as ${\hat{q}}_{\tau ,b}^{\left(k\right)}(x(t))$ .
Step 3. Given a sequence $\left\{\pi_{k}:\delta_{k}\in \Delta \right\}$ satisfying that $\pi_{k}\geq 0$ , $\sum_{\delta_{k}\in \Delta }\pi_{k}=1$ , use $N_{2}^{b}$ to calculate the b th exponential aggregation weights that ${\hat{\Omega }}_{k,N_{0}+1}^{b}=\pi_{k}$ and for $N_{0}+2\leq i\leq n$ ,
(3) $${\hat{\Omega }}_{k,i}^{b}=\frac{\pi_{k}\exp\;\left\{-\lambda \sum_{l=N_{0}+1}^{i-1}\sum_{j=1}^{n_{l}}{\hat{L}}_{\tau ,b,lj}^{\left(k\right)}\right\}}{\sum_{\delta_{k}\in \Delta }\pi_{k}\exp\;\left\{-\lambda \sum_{l=N_{0}+1}^{i-1}\sum_{j=1}^{n_{l}}{\hat{L}}_{\tau ,b,lj}^{\left(k\right)}\right\}}$$
  where ${\hat{L}}_{\tau ,b,lj}^{\left(k\right)}=\rho_{\tau ,h}^{*}(y_{l}^{b}(t_{lj})-{\hat{q}}_{\tau ,b}^{\left(k\right)}(x_{l}^{b}(t_{lj})))$ , λ>0 is the tuning parameter and $\rho_{\tau ,h}^{*}(v)$ is the secondary smoothing check loss function defined in (2).
Step 4. Repeat steps 1-3 by B times, the aggregated estimator of $q_{\tau }(x(t))$ is obtained by
$${\hat{q}}_{\tau }^{M}(x(t))=\frac{1}{B}\sum_{b=1}^{B}\sum_{i=N_{0}+1}^{n}\sum_{\delta_{k}\in \Delta }\frac{{\hat{\Omega }}_{k,i}^{b}}{n-N_{0}}{\hat{q}}_{\tau ,b}^{\left(k\right)}(x(t))$$

**Remark 1.** In addition to smoothing coefficients, there are several tuning parameters in Algorithm 1: λ controls the propensity of corresponding procedures in the weighting process; $\left\{\pi_{k}:k\geq 1\right\}$ are regarded as prior weights of candidates. In longitudinal quantile regression, the performance is similar to that in Shan and Yang,^21^ Gu and Zou^22^: λ→0 to average all of the candidate procedures and λ→∞ to select the best historic smoothing loss on the test set. Theoretically, we can set

$$\pi_{k}=\frac{\exp\;\left\{-\sum_{l=1}^{N_{0}}\sum_{j=1}^{n_{l}}{\hat{L}}_{\tau ,b,lj}^{\left(k\right)}\right\}}{\sum_{\delta_{k}\in \Delta }\exp\;\left\{-\sum_{l=1}^{N_{0}}\sum_{j=1}^{n_{l}}{\hat{L}}_{\tau ,b,lj}^{\left(k\right)}\right\}}$$

as a reasonable criterion and the upper-bound of λ will be exhibited in Section 3.1.

**Remark 2.** Although the EAW algorithm will be computationally intensive when the number of random partition is large, it eliminates the lack of accuracy due to randomly splitting sample. Indeed, multiple splits can be carried out in parallel as the results of each loop are mutually independent. All of the above will be shown during the theoretical proof and empirical studies. Besides, if the sequential updating mechanism is of less importance in practice, one can use the full-data set to implement Step 2 and to produce weights via split sets.

### 1.1. Estimating τ -CQF with censored history process

In this subsection, we evaluate τ -CQF for LQMM with right-censored history process. For the data in the form $\left(x_{i}^{⊤}(t_{ij}),y_{i}(t_{ij}),z_{i}^{⊤}(t_{ij}),t_{ij}\right)$ , $t_{ij}\in [0,T]$ , i=1,…,n , $j=1,\ldots ,n_{i}$ , we consider the additive mixed effect model whose τ th conditional quantile is:

(4) $$Q_{\tau }(y_{i}(t_{ij})|x_{i}(t_{ij}),z_{i}(t_{ij}),u_{i})=q_{F,\tau }(x_{i}(t_{ij}))+z_{i}^{⊤}(t_{ij})u_{i}$$

where $u_{i}$ is the q -dimensional subject-specific zero-mean random effects vector and $z_{i}(t_{ij})$ be an endogenous time-dependent covariates associated with $u_{i}$ . Generally, we assume $u_{i}=(u_{1,i},\ldots ,u_{q,i}{)}^{⊤}$ is independent of the model error term, identically distributed according to a determined density $f_{u}$ with zero-mean and a τ -dependent q×q covariance matrix $\Phi_{\tau }$ . Moreover, denote $z_{i}=(z_{i}(t_{i1}),\ldots ,z_{i}(t_{in_{i}}){)}^{⊤}$ , $y_{i}|x_{i},z_{i},u_{i}$ is supposed to be independently distributed with the joint asymmetric Laplace (AL) distribution, which provides a possible parametric link between minimizing the sum of check loss and maximizing the AL-likelihood (see in, Koenker and Machado,^25^ Geraci and Bottai^26^).

Note that $y_{i}|x_{i},z_{i},u_{i}∼\text{AL}(\mu ,\sigma ,\tau )$ has unknown parameters μ and σ , where the j th component of μ is $q_{F,\tau }(x_{i}(t_{ij}))+z_{i}^{⊤}(t_{ij})u_{i}$ , the joint density of $\left(y_{i},u_{i}\right)$ is

(5) $$f(y_{i},u_{i}|\mu ,\sigma ,\tau ,\Phi_{\tau })=f(y_{i}|u_{i},\mu ,\sigma ,\tau )f_{u}(u_{i}|\Phi_{\tau })$$

Fitting model (4) is equivalent to estimating parameters in (5). If $q_{F,\tau }(x_{i}(t_{ij}))$ is correctly specified, a number of methodologies have been proposed to make related inference including parametric methods (e.g. Geraci^12^) and nonparametric methods (e.g. Geraci^13^). As it cannot be determined under different conditions, the EAW algorithm is applicable for constructing the best approximation. Actually, let $\Delta =\{\delta_{k}:k=1,\ldots ,K\}$ be the set of total K -multiple candidate procedures, an aggregated estimator for the fixed effect part in equation (4) is

(6) $${\hat{q}}_{F,\tau }^{M}(x(t))=\frac{1}{B}\sum_{b=1}^{B}\sum_{i=N_{0}+1}^{n}\sum_{\delta_{k}\in \Delta }\frac{{\hat{\Omega }}_{k,i}^{b}}{n-N_{0}}{\hat{q}}_{F,\tau ,b}^{\left(k\right)}(x(t))$$

where ${\hat{q}}_{F,\tau ,b}^{\left(k\right)}(x(t))$ is the estimator by $\delta_{k}$ with the b th random split, ${\hat{\Omega }}_{k,i}^{b}$ is given in (3). Since the random effect part is specified as a known structure, the intention has been involved in ${\hat{q}}_{F,\tau ,b}^{\left(k\right)}(x(t))$ and it will not be added into weighting process.

For the i th subject, let $T_{i}$ and $C_{i}$ represent the terminal time and censoring time, respectively, and $\eta_{i}=I(T_{i}\leq C_{i})$ is the censoring indicator. $\left\{y_{i}(t_{ij}),t_{ij}\geq 0\right\}$ hence can be taken as observations of the history process that are ceased by the observed event time $T_{i}^{*}=\min\{C_{i},T_{i}\}$ . Our main interest is to estimate τ -CQF from the actual observations $\left\{(x_{i}^{⊤}(t_{ij}),y_{i}(t_{ij}),z_{i}^{⊤}(t_{ij}),t_{ij},\eta_{i}):i=1,\ldots ,n;j=1,\ldots ,n_{i}\right\}$ and $t_{in_{i}}\leq T_{i}^{*}$ .

Let $K_{1}(t)=\mathrm{Pr}(T_{i}>t)$ and $K_{2}(t)=\mathrm{Pr}(C_{i}>t)$ be the survival functions of $T_{i}$ , $C_{i}$ , and $K(t)=\mathrm{Pr}(T_{i}^{*}>t)$ be the survival function of actual cut-off time $T_{i}^{*}$ . Without loss of generality, we assume that censoring time is independent of terminal time and the history process for each subject. In the light of Liu et al.,^8^ the τ th quantile state function ( τ -QSF) of (4) at time t is defined as $H_{\tau }(t)=Q_{\tau }(y(t)|x(t),z(t),u)$ . We combine the inverse probability of censoring weighting (IPCW) method with EAW estimator equation (6) to estimate τ -QSF that

$${\hat{H}}_{\tau }(t)=\frac{1}{n}\sum_{i=1}^{n}\frac{I(T_{i}^{*}\geq t)}{\hat{K}(t)}{\hat{q}}_{F,\tau }^{M}(x_{i}(t))$$

where $\hat{K}(t)={\hat{K}}_{1}(t){\hat{K}}_{2}(t)$ , ${\hat{K}}_{l}(t)$ (l=1,2) are corresponding Kaplan–Meier estimators of $K_{1}(t)$ and $K_{2}(t)$ , respectively.

The τ -CQF in the time period [0,s] is defined as $\mu_{\tau }(s)=\int_{0}^{s}H_{\tau }(t)dt$ . Using the above estimator we can obtain that

(7) $${\hat{\mu }}_{\tau }(s)=\frac{1}{n}\sum_{i=1}^{n}\int_{0}^{s}\frac{I(T_{i}^{*}\geq t)}{{\hat{K}}_{1}(t){\hat{K}}_{2}(t)}{\hat{q}}_{F,\tau }^{M}(x_{i}(t))dt,\;s\in [0,L]$$

However, in practice, one can only observe $y_{i}(t)$ , covariate $x_{i}(t)$ at discrete time points $t_{ij},j=1,\ldots ,n_{i};i=1,\ldots ,n$ rather than in a continuous interval. Therefore, the linear interpolation in Deng^27^ is exploited to compute (7).

Let $D_{i}=\{t_{ij}:\max(t_{ij})\leq T_{i}^{*};j=0,1,\ldots ,n_{i}\}$ be the set of actual observed time points for the i th subject, and $t_{i0}=0$ means that observations of all subjects can be gathered in the initial time t=0 . We rearrange $t_{ij}$ for all subjects as $0=t_{\left(0\right)}<\cdots <t_{\left(N\right)}$ , and $D=\{t_{\left(k\right)},k=0,\ldots ,N\}$ is the set of ordered distinct observed time points. For $t_{\left(k\right)}\in D_{1}\cup \cdots \cup D_{n}$ , the fitted τ -QSF is given as

$${\hat{H}}_{\tau }(t_{\left(k\right)})=\frac{1}{n}\sum_{i=1}^{n}\frac{I(T_{i}^{*}\geq t_{\left(k\right)})}{{\hat{K}}_{1}(t_{\left(k\right)}){\hat{K}}_{2}(t_{\left(k\right)})}{\hat{q}}_{F,\tau }^{M}(x_{i}(t_{\left(k\right)}))$$

and for $t\in (t_{\left(k-1\right)},t_{\left(k\right)}]$ in a time quantum,

$${\hat{H}}_{\tau }(t)={\hat{H}}_{\tau }(t_{\left(k-1\right)})+\frac{t-t_{\left(k-1\right)}}{t_{\left(k\right)}-t_{\left(k-1\right)}}\left\{{\hat{H}}_{\tau }(t_{\left(k\right)})-{\hat{H}}_{\tau }(t_{\left(k-1\right)})\right\}$$

As a consequence, the estimator of τ -CQF at all observed time points $t_{\left(k\right)}\in D$ is

(8) $$\begin{array}{r} {\hat{\mu }}_{\tau }(t_{\left(k\right)})=\sum_{l=1}^{k}\int_{t_{\left(l-1\right)}}^{t_{\left(l\right)}}{\hat{H}}_{\tau }(t)dt=\frac{1}{2}\sum_{l=1}^{k}\left\{{\hat{H}}_{\tau }(t_{\left(l\right)})+{\hat{H}}_{\tau }(t_{\left(l-1\right)})\left(t_{\left(l\right)}-t_{\left(l-1\right)}\right)\right\} \end{array}$$

It is obviously shown that the estimation of (7) can be expressed by using $\left\{{\hat{\mu }}_{\tau }(t_{\left(k\right)}):k=1,\ldots ,N\right\}$ in (8). To be computational convenience for ${\hat{H}}_{\tau }(t)$ , we use a nonparametric approximation for ${\hat{K}}_{1}(t){\hat{K}}_{2}(t)$ by $n^{-1}\sum_{i=1}^{n}I(T_{i}^{*}\geq t)$ , and the estimators of $H_{\tau }(t)$ , $\mu_{\tau }(t)$ are reformed by ${\tilde{H}}_{\tau }(t)$ and ${\tilde{\mu }}_{\tau }(t)$ , replacing each ${\hat{H}}_{\tau }(t_{\left(k\right)})$ term with

(9) $${\tilde{H}}_{\tau }(t_{\left(k\right)})=\sum_{i=1}^{n}\frac{I(T_{i}^{*}\geq t_{\left(k\right)})}{\sum_{j=1}^{n}I(T_{j}^{*}\geq t_{\left(k\right)})}{\hat{q}}_{F,\tau }^{M}(x_{i}(t_{\left(k\right)}))$$

### 1.2. EAW-based prediction for random effects

Predicting random effects for LQMM is an ongoing research issue. Geraci and Bottai^26^ proposed a best linear prediction (BLP) of $u_{i}$ in terms of linear fixed effect. For a given probability level, the predictor depends on the estimation of parameters in (5) with correct specification of $q_{F,\tau }(x_{i}(t_{ij}))$ and the construction of $u_{i}$ , and satisfies that its mean squared error (MSE) reaches the minimum. In (4) we assume that random effects have a linear pattern $z_{i}^{⊤}(t_{ij})u_{i}$ , for the i th cluster, the BLP of $u_{i}$ is given as

$${\hat{u}}_{i}^{\left(\tau \right)}={\hat{\Phi }}_{\tau }z_{i}^{⊤}{\hat{\Sigma }}_{i}^{-1}(y_{i}-\hat{E}y_{i})$$

where $\Sigma_{i}=z_{i}\Phi_{\tau }z_{i}^{⊤}+{\text{Cov}}_{i}$ and ${\text{Cov}}_{i}$ is regarded as the covariance matrix of random errors in (4). However, since diverse procedures result in changeable estimators, the main purpose of this subsection is to find the most effective prediction. A natural thought is to utilize the exponential aggregated weights to contribute the predictor among candidate $q_{F,\tau }(x(t))$ .

As we mentioned previously, candidate predictors can be generated with a full-data set as long as we ignore the sequential updating mechanism. It is suitable since the predictor may be lack of efficiency if we only use a fraction of the samples to predict all random effects. Let ${\hat{u}}_{i}^{(\tau ),k}$ be the BLP of the procedure $\delta_{k}$ with respect to the i th cluster, and ${\hat{\Omega }}_{k,m}^{b}$ $\left(N_{0}+1\leq m\leq n\right)$ be the exponential aggregated weights from Algorithm 1. Denote that

$${\hat{\Omega }}_{k}=\frac{1}{B}\sum_{b=1}^{B}\sum_{m=N_{0}+1}^{n}\frac{{\hat{\Omega }}_{k,m}^{b}}{n-N_{0}}$$

Then the EAW-based BLP of the random effects can be computed as

(10) $${\hat{u}}_{i}^{(\tau ),M}=\sum_{\delta_{k}\in \Delta }{\hat{\Omega }}_{k}{\hat{u}}_{i}^{(\tau ),k},\;i=1,\ldots ,n$$

## 2. Asymptotic properties

In this section, we build the asymptotic properties of proposed estimators. The outperformance of EAW estimator is critiqued by the oracle inequalities to show that the risks are automatically close to those of the best candidate. By the secondary smoothing loss function (2), we derive a homologous risk bound under quadratic losses. On the other hand, the consistency of the proposed τ -CQF is given as long as the candidate set involves a consistent procedure, which is conveniently verified under the smoothing strategy.

### 2.1. Oracle inequalities

We illustrate the theoretical properties of EAW estimator of equation (1). The intended adaptivity of the estimator is emerged via oracle inequalities, which provide statistical risk bounds in the sense of original check loss and squared error loss, respectively. For longitudinal data, risk bounds depend on the formulation of data collection as well. Define the counting process of time points for observations on the i th subject as $N_{i}(t)=\sum_{j=1}^{n_{i}}I(t_{ij}\leq t)$ , we refer to Fan and Li^28^ to assume $N_{i}(t)$ to be a random sample from a certain population in the finite interval. Moreover, let the $L_{2}$ norm of a generic function f with respect to the distribution $P_{X}$ of X be $\|f{\|}^{2}=\int f^{2}(x)dP_{X}$ . Following conditions are imposed for theoretical results.

(A.1)
$\underset{\delta_{k}\in \Delta }{\sup}\max_{t\in [0,T]}|{\hat{q}}_{\tau }^{\left(k\right)}(x(t))-q_{\tau }(x(t))|\leq C_{\tau }$ with probability one.
(A.2) Let $e_{\tau }(t)=y(t)-q_{\tau }(x(t))$ , the $\max_{t}$ -sub-exponential norm of $e_{\tau }(t)$ given x(t) and t is bounded by $K_{\tau }$ , where the $\max_{t}$ -sub-exponential norm of X(t) is defined as
$$\max_{t}\|X(t){\|}_{\text{SEXP}}:=\max_{t\in [0,T]}\left\{\underset{p\geq 1}{\sup}p^{-1}{\left(E|X(t){|}^{p}\right)}^{1/p}\right\}$$
  Further, there exists a positive $K_{\rho }^{{}^{\prime }}$ such that $\int_{-K_{\tau }}^{K_{\tau }}|\rho_{h,\tau }^{*}(v)-\rho_{\tau }(v)|dv\leq K_{\rho }^{{}^{\prime }}$ .

Condition (A.1) and the upper-boundedness of the sub-exponential norm are fairly common in the related work of aggregations. The constraints are mild to be satisfied only if $q_{\tau }(x(t))$ and the variance of $e_{\tau }(t)$ are bounded almost surely. In addition, the boundedness of the integral of $|\rho_{h,\tau }^{*}(K_{\tau })-\rho_{\tau }(K_{\tau })|$ on $\left[-K_{\tau },K_{\tau }\right]$ is equivalent to the upper-boundedness of a(h)h , which implies that $\rho_{\tau ,h}^{*}$ performs close to $\rho_{\tau }$ . Based on the above conditions, it can be shown that the EAW estimator behaves optimally with the best procedure via the following theorem:

Theorem 1 Under conditions (A.1) – (A.2) , if λ satisfies

$$\lambda \leq \min\left\{\frac{\underline{c}\exp\;(-2\bar{c}C_{\tau }(4e{\bar{K}}_{\zeta }{)}^{-1})}{\left(16\sqrt{2}T^{2}K_{\tau }^{2}M_{0}+16{\bar{c}}^{2}TC_{\tau }^{2}M_{0}\right)},\frac{(2e{)}^{-1}}{4TC_{\tau }{\bar{K}}_{\zeta }}\right\}$$

the risk of EAW estimator under the original check loss $\rho_{\tau }(\cdot )$ satisfies

(11) $$\begin{array}{r} E\int \rho_{\tau }\left(y-{\hat{q}}_{\tau }^{M}(x(t))\right)dN(t)\leq \underset{\delta_{k}\in \Delta }{\inf}\left\{E\int \rho_{\tau }\left(y-{\hat{q}}_{\tau }^{\left(k\right)}(x(t))\right)dN(t)+\frac{\log\;(1/\pi_{k})}{\lambda (n-N_{0})}+H_{1}\right\} \end{array}$$

Moreover, the risk of EAW estimator under the squared error loss satisfies

(12) $$\begin{array}{r} E\int {\left\|{\hat{q}}_{\tau }^{M}(x(t))-q_{\tau }(x(t))\right\|}^{2}dN(t)\leq \underset{\delta_{k}\in \Delta }{\inf}\left\{\frac{\bar{c}}{\underline{c}}E\int {\left\|{\hat{q}}_{\tau }^{\left(k\right)}(x(t))-q_{\tau }(x(t))\right\|}^{2}dN(t)+\frac{\log\;(1/\pi_{k})}{\underline{c}\lambda (n-N_{0})}\right\} \end{array}$$

where $M_{0}=\exp\;\{(4T{\bar{K}}_{\zeta }{)}^{-1}+(4e{)}^{-1}\}$ , $H_{1}$ , ${\bar{K}}_{\zeta }$ , $\underline{c}$ and $\bar{c}$ are positive, which will be presented in the proof.

**Remark 3.** Through the proof we have $H_{1}=Th\max\{\tau^{2},(1-\tau^{2})\}$ , the risk bound under the original check loss is unavoidably dominated by the smoothing parameter as the aggregated weights are contributed via the secondary smoothing loss function. On the other hand, the smoothing parameter h is restricted by $K_{\rho }^{{}^{\prime }}$ so that it cannot be too large. When h is chosen near zero, the EAW estimator has an approximate performance with that by original loss. Comparing with Shan and Yang,^21^ we can find that the converge rate of EAW estimator is fast as long as $H_{1}+\log\;(1/\pi_{k})/\{(\lambda (n-N_{0})\}=o((n-N_{0}{)}^{-1/2})$ . In practice if we only care about the prediction risk, one can use $h=(n-N_{0}{)}^{-1}$ , $a(h)=n^{-1}h$ to fit a drastic transition of ${\hat{e}}_{\tau }^{\left(k\right)}(t)$ .

**Remark 4.** The second inequality in Theorem 1 should not have been obtained by the original loss of quantile regression. However, it provides an advantageous demonstration for the asymptotic properties of τ -CQF estimator. From a model specification perspective, (12) shows that the EAW estimator has an analogous consistency as long as Δ contains the correct candidate. This requires that $\bar{c}/\underline{c}$ is upper-bounded theoretically, which is by no means mutually exclusive with Condition (A.2).

### 2.2. Consistency for τ -CQF estimator

In this subsection, we anchor our investigation to demonstrate the consistency of estimators in Section 2.2. Let M(t)={x(t),z(t)} denote the observed covariate process such as baseline information and study time and so on, ${\bar{B}}_{Y}(t)=\{Y(s):s<t\}$ and ${\bar{B}}_{M}(t)=\{M(s):s<t\}$ be the longitudinal response history and the longitudinal covariate history prior to time t . A set of regularity conditions are given to establish the theoretical property.

(B.1) Conditional on u , ${\bar{B}}_{M}(t)$ , ${\bar{B}}_{Y}(t)$ and T≥t , M(t) is fully observed and its distribution depends only on ${\bar{B}}_{M}(t)$ for t∈[0,L] . In addition, M(t) is continuously differentiable in [0,L] with probability one and $\max_{t\in [0,L]}\|M^{{}^{\prime }}(t)\|<\infty$ , where $M^{{}^{\prime }}(t)$ denotes the derivative of M(t) at time t .
(B.2) For any t<L , the intensity of the counting process $N_{C}(t)$ given u , ${\bar{B}}_{M}(t)$ , ${\bar{B}}_{Y}(t)$ and T≥t is determined only by ${\bar{B}}_{M}(t)$ and M(t) , where $N_{C}(t)=\sum_{i=1}^{C}I(t_{i}\leq t)$ .
(B.3) For $\delta_{k}\in \Delta$ , there exits the nonstochastic function ${\bar{q}}_{F,\tau }^{\left(k\right)}(x(t))$ such that for t∈[0,T] , $\left\|{\hat{q}}_{F,\tau }^{\left(k\right)}(x(t))-{\bar{q}}_{F,\tau }^{\left(k\right)}(x(t))\|=o_{p}(1\right)$ . Besides, $\left\|{\hat{\Phi }}_{\tau }^{\left(k\right)}-{\bar{\Phi }}_{\tau }^{\left(k\right)}\|=o_{p}(1\right)$ with some ${\bar{\Phi }}_{\tau }^{\left(k\right)}$ in the support of $\Phi_{\tau }$ .
(B.4) Given a positive constant $M_{0}$ , $\max_{t\in [0,T]}\|{\bar{q}}_{F,\tau }^{\left(k\right)}(x(t))\|\leq M_{0}$ and $\min_{\|b\|=1}b^{⊤}{\bar{\Phi }}_{\tau }^{\left(k\right)}b>M_{0}^{-1}$ for $\delta_{k}\in \Delta$ .
(B.5) There exists a constant a>0 such that $\min\{K_{1}(L),K_{2}(L)\}\geq a$ .
(B.6) There exits some $K_{\rho }^{{}^{\prime \prime }}>0$ such that $a(h)h\geq K_{\rho }^{{}^{\prime \prime }}$ .

Aforementioned assumptions are widely considered in Zeng and Cai,^29^ Deng,^27^ and Liu et al.,^8^ which are essential to longitudinal time-to-event data with censoring mechanism. Condition (B.3) is typically recommended to respond the misspecification of $q_{F,\tau }(x(t))$ , and guarantees the convergency of candidate procedures as well. Condition (B.6), combined with Condition (A.2), claims that the smoothing coefficients of $\rho_{\tau ,h}^{*}(v)$ are controlled in an intercell near the right side of the origin to guarantee both the approximation of $\rho_{\tau }(v)$ and the consistency of τ -QSF. Based on the above conditions, we establish the consistency of the estimators for τ -QSF, τ -CQF by the following theorem:

Theorem 2 Suppose that Conditions (A.1) – (A.2) and (B.1) – (B.6) hold, for a given probability level 0<τ<1 , if ${\bar{q}}_{F,\tau }^{\left(k\right)}(x(t))=q_{F,\tau }(x(t))$ for some $\delta_{k}\in \Delta$ , ${\hat{H}}_{\tau }(t)$ is a consistent estimator of τ -QSF, and ${\hat{\mu }}_{\tau }(s)$ in (7) is consistent to $\mu_{\tau }(s)$ .

**Remark 5.** Theorem 2 implies that the consistency of τ -CQF estimator is constructed by the effective estimation of the fixed effect, i.e. Δ contains the correct specification of $q_{F,\tau }(x(t))$ . Instead of predicting random effects, the constraint of model (4) can be changed to degenerate into a classical case for the particular practice. Traditional quantile loss estimation may be considered as one of the candidate procedure. See Galvao et al.^6^, Harding and Lamarche^7^ for corresponding discussions.

It is worthily shown that the same construction of Theorem 2 will be derived in terms of ${\tilde{\mu }}_{\tau }(s)$ and ${\tilde{H}}_{\tau }(t)$ , which performs much more popular for handling practical problems.

## 3. Numerical studies

### 3.1. Simulation

In this section, we report the performance of EAW estimators proposed in previous sections by Monte Carlo simulation studies for longitudinal quantile regression. We firstly design an experiment for equation (1), then fit τ -QSF and τ -CQF for mixed effect model.

**Experiment 1.** In this experiment, we consider a general longitudinal quantile model as follows:

(13) $$\left\{ \begin{array}{rl} & y_{i}(t_{ij})=q_{1,\tau }+q_{2,\tau }+q_{3,\tau }\varepsilon_{i}(t_{ij})\\ & q_{1,\tau }=\beta_{1,\tau }x_{1,i}(t_{ij})+\beta_{3,\tau }x_{3,i}\\ & q_{2,\tau }=(0.5-\tau )\left\{\alpha_{1,\tau }(t_{ij})x_{1,i}(t_{ij})+\alpha_{2,\tau }(t_{ij})x_{2,i}\right\}\\ & q_{3,\tau }=(0.75-\tau )\left\{f_{2,\tau }(x_{2,i})+f_{3,\tau }(x_{3,i})\right\} \end{array}\right.$$

For τ∈(0,1) , model (13) is composed of the constant coefficient linear part $q_{1,\tau }$ , the varying-coefficient part $q_{2,\tau }$ and the nonparametric part $q_{3,\tau }$ . The observation time points for each subject value in the scheduled set {1,…,[T]} and [T] is the terminal time satisfying T∼U(10,30) . The random errors $\varepsilon_{i}=(\varepsilon_{i}(t_{i1}),\ldots ,\varepsilon_{i}(t_{in_{i}}){)}^{⊤}$ are generated from a multivariate normal distribution with mean $0_{1\times n_{i}}$ and an AR(1) structure variance-covariance matrix

$$\Sigma_{n_{i}}(\rho )=\left(\begin{array}{cccc} 1 & \rho & \cdots & \rho^{n_{i}-1}\\ \rho & 1 & \cdots & \rho^{n_{i}-2}\\ \vdots & \vdots & \ddots & \vdots \\ \rho^{n_{i}-1} & \rho^{n_{i}-2} & \cdots & 1 \end{array}\right)$$

where ρ=0.5 to make the term with medium correlation. Besides, the covariates $x_{1,i}(t)∼U(t/20,2+t/20)$ ,

$$\left(\begin{array}{c} x_{2,i}\\ x_{3,i} \end{array}\right)∼N\left(\left(\begin{array}{c} 1\\ 0 \end{array}\right),\left(\begin{array}{cc} 1 & \left[T\right]/35\\ \left[T\right]/35 & 1 \end{array}\right)\right)$$

and $\left(\beta_{1,\tau },\beta_{3,\tau })=(0.5,-1\right)$ . The varying-coefficients $\alpha_{1,\tau }(t)=0.8\sin\;(\pi t/20)$ , $\alpha_{2,\tau }(t)=3-\cos\;((t-25)\pi /15)$ , and the nonparametric form $f_{2,\tau }(x)=(2x-1)/(x^{2}+1)$ , $f_{3,\tau }(x)=\exp\;(0.5x+1)$ , respectively.

According to the above specification, the τ th conditional quantile function is presented as $Q_{\tau }(y(t)|x(t))=q_{1,\tau }+q_{2,\tau }+q_{3,\tau }b_{\tau }$ , where $b_{\tau }$ is the τ -quantile of N(0,1) . Intuitively, under particular quantile levels, different terms dominate the performance of the true model (such as when τ=0.5 , $Q_{\tau }(y(t)|x(t))=q_{1,\tau }$ and the varying-coefficient regression approximates the model better as τ near 0.75 , among others). To compare EAW estimator with a single procedure, we construct three candidate models to fit (13):

1. LQR:
$$Q_{1,\tau }(y(t)|x(t))=\theta_{1}x_{1}(t)+\theta_{2}x_{2}+\theta_{3}x_{3}$$
2. Vary-Coefficient LQR (VCLQR):
$$Q_{2,\tau }(y(t)|x(t))=\theta_{1}(t)x_{1}(t)+\theta_{2}(t)x_{2}+\theta_{3}(t)x_{3}$$
3. Nonprametric additive quantile regression (NAQR):
$$Q_{3,\tau }(y(t)|x(t))=\theta_{1}(x_{1}(t))+\theta_{2}(x_{2})+\theta_{3}(x_{3})$$

We use the LQR for estimating LQR, and B-spline estimations for VCLQR and NAQR. We take the size of subjects as n=200 with 500 independent replications. In the procedure of EAW, all subjects are randomly partitioned into a training set of $N_{0}=n/2$ and the test set for others, $\pi_{k}=1/3$ for k=1,2,3 and set the tuning parameter λ=1 . The algorithm is averaged over B=50 random splits. Moreover, smoothing parameters of the secondary smoothing loss function are set up as h=1 and a(h)=0.01 . Denote the estimation of the true quantile function $Q_{\tau }$ as ${\hat{Q}}_{\tau }$ from a specific procedure. The estimated (original check loss based) prediction risk (PR) and the estimated root MSE (RMSE) are designed as quantization criteria that

$$\begin{array}{rl} & \text{PR}(\tau )=\frac{1}{n}\sum_{i=1}^{n}\frac{1}{n_{i}}\sum_{j=1}^{n_{i}}\rho_{\tau }\left(y_{i}(t_{ij})-{\hat{Q}}_{\tau ,ij}\right)\\ & \text{RMSE}(\tau )=\sqrt{\frac{1}{n}\sum_{i=1}^{n}\frac{1}{n_{i}}\sum_{j=1}^{n_{i}}{\left({\hat{Q}}_{\tau ,ij}-Q_{\tau ,ij}\right)}^{2}} \end{array}$$

**Figure 2.**
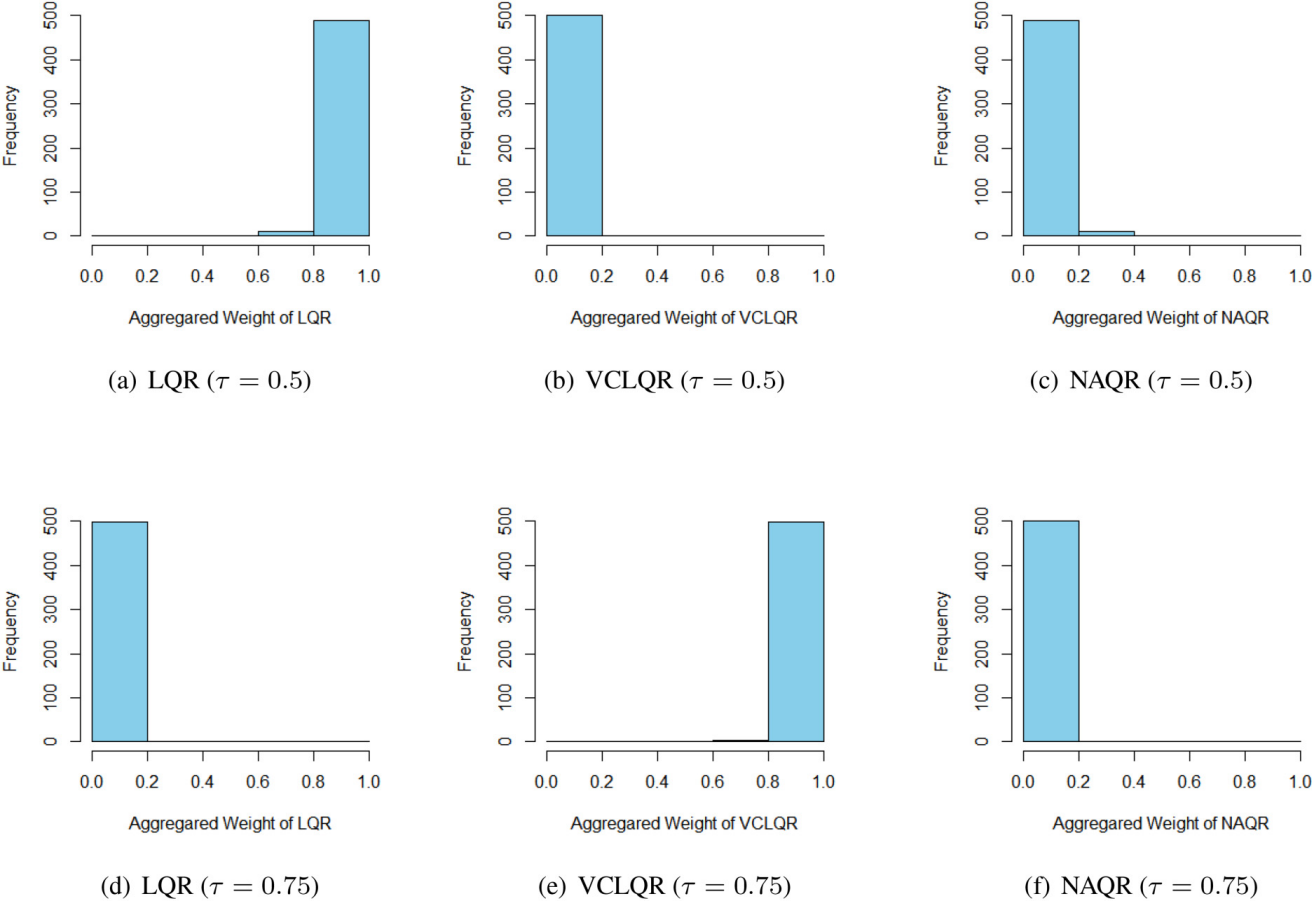
Histograms of exponential aggregation weights of candidate procedures for either τ=0.5 or 0.75 under 200 sample size and 500 replications.

Table 1 reports the performance measures for all candidate procedures and EAW estimator under different probability levels and thickens the best among candidates. One can notice when τ<0.5 the nonlinear form $q_{3,\tau }$ plays a leading role and NAQR fits the model better than LQR and VCLQR. Conversely, when τ is larger than 0.75, VCLQR is more suitable for the specified model. As τ tends to the median, three kinds of specifications result in the satisfying PR but the LQR model has a smaller RMSE than the other two nonparametric estimators. All the above results are fundamentally consistent with our prediction. Figure 2 typically presents the frequency of the value of aggregated weights among computations, which indicates that our weights are chosen around the best candidate and, the PR and RMSE of the EAW estimator emerge close to those of the “most approximate” specification of the true model under different probability levels. This phenomenon confirms the outperformance of our adaptive aggregation method in the longitudinal quantile model. The number in brackets represents the standard error of corresponding indicators, which shows that the EAW estimator can keep a stable deviation if one of the above candidates leads an effective result.

To demonstrate the rationality of the methodology in Section 2.2, we conduct the following simulation.

**Experiment 2.** Assume the additive mixed effect model (4) has the following structure:

(14) $$y(t)=\beta_{1,\tau }t+\beta_{2,\tau }x_{2}+(\tau -0.5)\alpha_{\tau }(t)+zu_{1}+u_{2}+f_{\tau }(x_{2})\varepsilon$$

**Table 1.** PRs, RMSEs, and related SEs (in parentheses) for kinds of estimations of Experiment 1, with the correlation coefficient ρ=0.5 of random errors. All results are multiplied by $10^{2}$ .

	Measure	Procedure			
τ	( $\times 10^{2}$ )	LQR	VCLQR	NAQR	Aggregation
0.05	PR	27.94(0.98)	28.16(1.19)	**24**.**18(0.62)**	25.26(1.17)
	RMSE	165.59(**8.89**)	166.03(11.18)	**91**.**10**(9.64)	114.10(20.79)
0.1	PR	41.50(1.02)	41.02(1.14)	**37**.**95(0.84)**	38.60(1.13)
	RMSE	116.79(**4.30**)	115.14(6.71)	**73**.**66**(7.49)	75.47(10.23)
0.25	PR	53.34(1.13)	53.57(1.14)	**51**.**82(1.10)**	51.77(1.08)
	RMSE	52.96(**1.46**)	59.46(2.18)	**42**.**22**(3.86)	38.55(3.10)
0.4	PR	43.47(**0.90**)	46.20(0.97)	**43**.**37**(0.91)	43.31(0.90)
	RMSE	**18**.**00(0.96)**	44.72(2.93)	20.74(2.91)	16.66(1.42)
0.5	PR	**31**.**60(0.62)**	35.92(0.79)	32.10(0.64)	31.60(0.62)
	RMSE	**2**.**18(1.36)**	42.04(3.20)	14.38(2.43)	2.37(1.31)
0.75	PR	11.86(0.94)	**8**.**88(0.58)**	12.98(1.00)	8.69(0.58)
	RMSE	**44**.**02**(2.80)	44.73(**1.67**)	48.58(3.14)	43.14(1.64)
0.9	PR	14.63(0.74)	**14**.**58(0.57)**	15.14(0.84)	14.29(0.57)
	RMSE	196.81(8.10)	**152**.**37(2.78)**	201.72(7.63)	153.34(2.90)
0.95	PR	**10**.**65(0.42)**	12.42(0.50)	10.86(0.56)	11.49(0.78)
	RMSE	308.83(11.03)	**254**.**33(5.16)**	317.35(10.55)	259.91(7.53)

Note: The best indicator among candidates are presented in bold. RMSE: root mean squared error; LQR: linear quantile regression; VCLQR: vary-coefficient linear quantile regression; NAQR: nonprametric additive quantile regression; PR: prediction risk.

we take $x_{2}$ be independently distributed from $\chi_{1}^{2}$ and $z=\xi_{1}+\xi_{2}$ , $\xi_{1}∼N(0,1)$ and $\xi_{2}∼N(0,1)$ . $\left(\beta_{1,\tau },\beta_{2,\tau }\right)$ are parameters of fixed effect and valued as (0.5,0.5) , $\alpha_{\tau }(t)=3\sin\;(\pi (t-10)/15)$ and $f_{\tau }(x_{2})=\exp\;(-0.3x_{2}+1)$ , respectively. The random effects are generated as

$$\left(\begin{array}{c} u_{1,i}\\ u_{2,i} \end{array}\right)∼N\left(0,\left(\begin{array}{cc} 4 & 0\\ 0 & 4 \end{array}\right)\right)$$

and the random error ε has the τ th quantile $b_{\tau }$ , of which distribution is set up as either standard normal distribution N(0,1) or standard Laplace distribution Lap(0,1) .

To specify different observed times of an unbalanced design, we utilize the following hazard function to generate the survival time T :

$$h(t)=h_{0}\exp\;\{w\gamma +\alpha \left(\beta_{1,\tau }t+\beta_{2,\tau }x_{2}\right)\}$$

Besides, the censoring point C can be sampled from a uniform distribution $U(a_{C},b_{C})$ . For each subject, the process with censored observations in (14) is similar to Deng,^27^ where the lifetime random sample $\left\{v_{i}:i=1,\ldots ,n\right\}$ with h(t) is generated via the following expression:

$$v_{i}=\frac{1}{\alpha \beta_{1,\tau }}\log\;\left\{1-\frac{\alpha \beta_{1,\tau }\log\;(1-s_{i})}{h_{0}\exp\;(w\gamma +\alpha \beta_{2,\tau }x_{2,i})}\right\}$$

where α=0.25 , $h_{0}=2\times 10^{-3}$ , w∼U(0,1) , γ=1 , and $s_{i}∼U(0,1)$ . Set the simulated terminal time $t_{i}=\min\{v_{i},c_{i}\}$ that controls the censoring rate at about 32% via $a_{C}=20$ and $b_{C}=40$ . Hence, for a probability level τ , the right-censored longitudinal observations are generated by

(15) $$y_{i}(s)=\beta_{1,\tau }s+\beta_{2,\tau }x_{2,i}+(\tau -0.5)\alpha_{\tau }(s)+z_{i}u_{1,i}+u_{2,i}+f_{\tau }(x_{2,i})\varepsilon_{i}$$

where i=1,…,n and $s=1,\ldots ,\lceil t_{i}\rceil$ . We specify LQR and NAQR with constant coefficients to fit the fixed effect part in (15) by using the “LQMM” package, and supply a misspecified linear quantile model (MLQR) $Q_{\tau }(y_{i}(t)|x_{i}(t),z_{i}(t),u_{i},t)=\beta_{1,\tau }\sqrt{t}+(\beta_{2,\tau }+u_{1,i})z_{i}+\beta_{3,\tau }+u_{2,i}$ for the median case. The EAW algorithm is adopted to combine candidate models and to estimate τ -QSF, τ -CQF, respectively. The simulation studies are carried out by 500 successful completions of algorithm. Simple computation leads that the “true function” of $H_{\tau }(t)$ and $\mu_{\tau }(t)$ are

$$\begin{array}{rl} H_{\tau }(t) & =0.5t+(3\tau -1.5)\sin\;\left(\frac{\pi (t-10)}{15}\right)+M_{f}b_{\tau }+0.5\\ \mu_{\tau }(t) & =0.25t^{2}-\frac{\left(45\tau -22.5\right)}{\pi }\cos\;\left(\frac{\pi (t-10)}{15}\right)+(M_{f}b_{\tau }+0.5)t \end{array}$$

where $M_{f}$ is averaged by the sample of $f_{\tau }(x_{2,i})$ generated among simulations.

**Figure 3.**
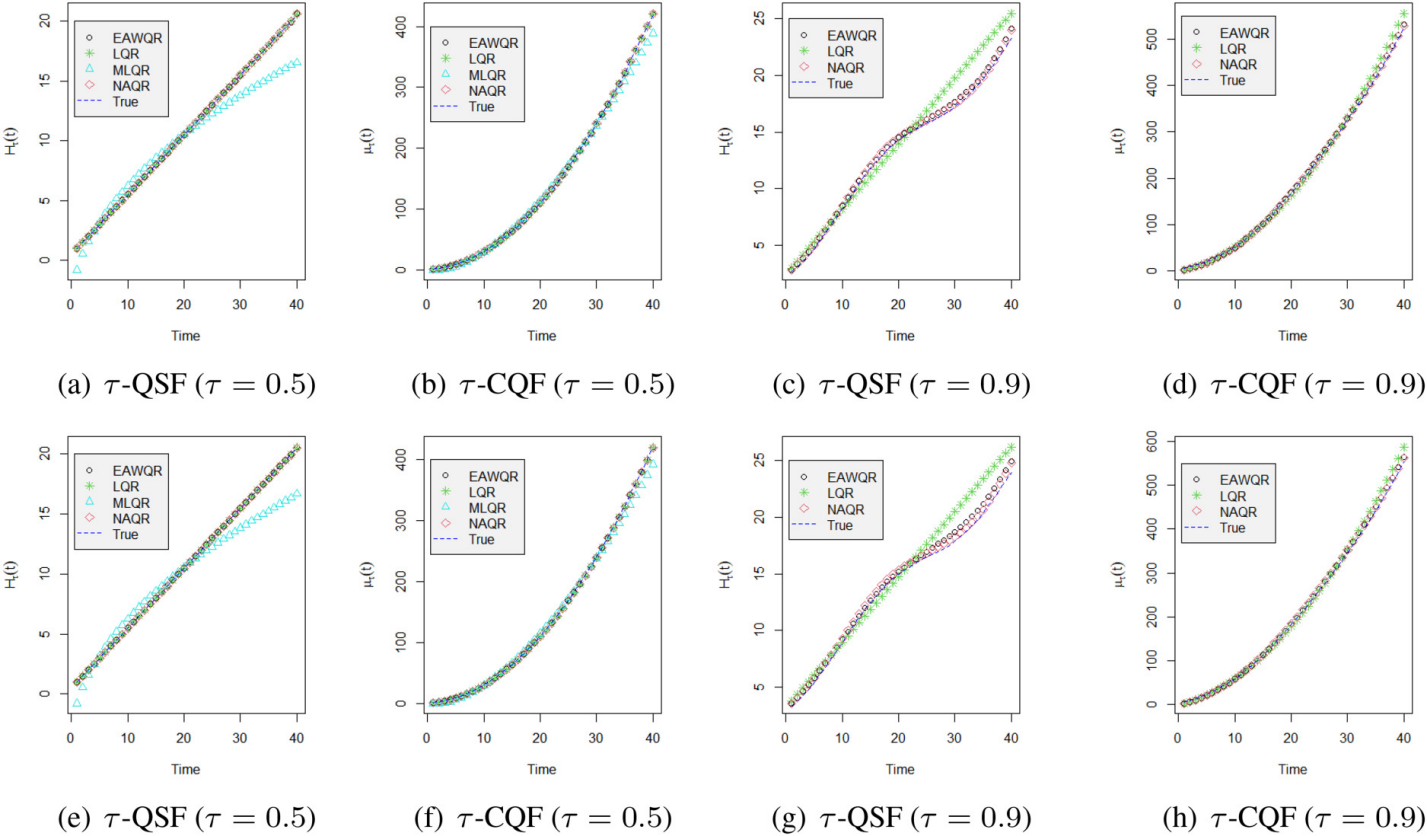
Fitted values versus the true curve of τ -QSF, τ -CQF for either τ=0.5 or 0.9 under different specifications with 300 subjects. The first row shows fitted curves with standard normal errors, and the second row is obtained under Laplace errors.

We implement the aforementioned experiment with sample size n=120 and 300 , split 2/3 of the total subjects for training and other 1/3 for testing. Smoothing parameters of the secondary smoothing loss function are taken with the same value as those in Experiment 1, and the weighting process is carried out with a single partition. We present the estimated PR and RMSE of the fixed effect part in Tables 2 and 3. Moreover, the measure of estimators for $H_{\tau }(t)$ and $\mu_{\tau }(t)$ is displayed as average MSE (AMSE) that

$$\text{AMSE}=\frac{1}{TR}\sum_{r=1}^{R}\int_{0}^{T}{\left\{{\hat{f}}^{\left(r\right)}(t)-f(t)\right\}}^{2}dt$$

where ${\hat{f}}^{\left(r\right)}(t)$ is the corresponding estimated function of f(t) in the r th replication. It can be seen that in most cases the aggregated estimator shows the gratifying performance regardless of the change of τ and ε . The PR and RMSE of the fixed effect emerge the same conclusion as those in Experiment 1. Note that model (15) approaches to the linear structure for τ=0.5 , and the nonparametric specification leads a more appreciable result otherwise. Although the split for the aggregation is set by B=1 , the EAW estimator still leads to expected properties and hence is verified to be feasible. For the estimator of τ -QSF and τ -CQF, we can find that the performance of AMSEs is consistent with that of PRs and RMSEs in different specifications: When τ=0.5 , LQR has smaller AMSEs than NAQR while latter fits the model much more conveniently otherwise, and the EAW estimator preserves an excellent accuracy in all cases. Figure 3 visualizes the above conclusions, whose tails of fitted curves are partly biased since the number of observed subjects decreases due to the failure of observations and censoring. Particularly, for median, we supply an additional misspecified mixed effect model MLQR to make the comparison. The results show that our EAW estimator can effectively distinguish the incorrect model as well.

**Table 2.** Fitting the fixed effect of Experiment 2, estimating τ -QSF and τ -CQF with different candidate procedures, including the incorrect specification, where ε∼N(0,1) .

			Procedure			
n	τ	Measure	LQR	MLQR	NAQR	Aggregation
120	0.25	PR	1.404(0.066)	–	**1**.**322(0.066)**	1.335(0.067)
		RMSE	0.767(0.200)	–	**0**.**410(0.199)**	0.466(0.198)
		${\text{AMSE}}_{H(t)}$	0.869	–	**0**.**403**	0.507
		${\text{AMSE}}_{\mu (t)}$	179.941	–	**74**.**138**	86.788
	0.5	PR	**1**.**580(0.057)**	1.648(0.059)	1.582(0.058)	1.581(0.058)
		RMSE	**0**.**212(0.130)**	1.210(0.147)	0.354(0.171)	0.295(0.183)
		${\text{AMSE}}_{H(t)}$	**0**.**120**	2.202	0.377	0.227
		${\text{AMSE}}_{\mu (t)}$	**30**.**061**	105.668	62.796	38.539
	0.9	PR	0.950(0.082)	–	**0**.**829(0.059)**	0.844(0.061)
		RMSE	1.010(**0.188**)	–	**0**.**579**(0.201)	0.615(0.190)
		${\text{AMSE}}_{H(t)}$	2.356	–	**0**.**420**	0.615
		${\text{AMSE}}_{\mu (t)}$	211.733	–	**81**.**527**	86.382
300	0.25	PR	1.364(0.045)	–	**1**.**321(0.042)**	1.324(0.043)
		RMSE	0.581(**0.113**)	–	**0**.**300**(0.145)	0.334(0.146)
		${\text{AMSE}}_{H(t)}$	0.780	–	**0**.**224**	0.283
		${\text{AMSE}}_{\mu (t)}$	71.095	–	**40**.**393**	45.674
	0.5	PR	**1**.**577(0.036)**	1.644(0.037)	1.579(0.036)	1.578(0.036)
		RMSE	**0**.**147**(0.102)	1.153(**0.096**)	0.248(0.140)	0.190 (0.118)
		${\text{AMSE}}_{H(t)}$	**0**.**070**	2.414	0.189	0.121
		${\text{AMSE}}_{\mu (t)}$	**16**.**696**	101.240	36.221	22.317
	0.9	PR	0.862(**0.041**)	–	**0**.**836**(0.043)	0.836(0.040)
		RMSE	0.875(**0.078**)	–	**0**.**431**(0.165)	0.483(0.161)
		${\text{AMSE}}_{H(t)}$	2.643	–	**0**.**237**	0.375
		${\text{AMSE}}_{\mu (t)}$	149.028	–	**47**.**922**	54.292

Note: The best indicator among candidates are presented in bold. RMSE: root mean squared error; LQR: linear quantile regression; NAQR: nonprametric additive quantile regression; MLQR: misspecified linear quantile model; PR: prediction risk; AMSE: average mean squared error; AMSE: average mean squared error.

**Table 3.** Fitting the fixed effect of Experiment 2, estimating τ -QSF and τ -CQF with different candidate procedures, including the incorrect specification, where ε∼Lap(0,1) .

			Procedure			
n	τ	Measure	LQR	MLQR	NAQR	Aggregation
120	0.25	PR	1.551(0.064)	–	**1**.**475(0.054)**	1.493(0.059)
		RMSE	0.672(**0.164**)	–	**0**.**477**(0.215)	0.494(0.185)
		${\text{AMSE}}_{H(t)}$	0.928	–	**0**.**526**	0.655
		${\text{AMSE}}_{\mu (t)}$	114.439	–	**85**.**878**	82.199
	0.5	PR	**1**.**759(0.061)**	1.821(0.061)	1.760(0.062)	1.760(0.061)
		RMSE	**0**.**210(0.137)**	1.202(0.141)	0.350(0.169)	0.286(0.163)
		${\text{AMSE}}_{H(t)}$	**0**.**121**	2.148	0.376	0.226
		${\text{AMSE}}_{\mu (t)}$	**30**.**418**	100.972	58.478	37.915
	0.9	PR	0.986(0.069)	–	**0**.**901(0.048)**	0.926(0.054)
		RMSE	1.048(**0.170**)	–	**0**.**716**(0.210)	0.767(0.198)
		${\text{AMSE}}_{H(t)}$	2.593	–	**0**.**576**	1.008
		${\text{AMSE}}_{\mu (t)}$	195.698	–	**100**.**184**	98.928
300	0.25	PR	1.514(0.041)	–	**1**.**480(0.038)**	1.484(0.038)
		RMSE	0.542(**0.069**)	–	**0**.**358**(0.157)	0.379(0.143)
		${\text{AMSE}}_{H(t)}$	0.924	–	**0**.**295**	0.406
		${\text{AMSE}}_{\mu (t)}$	58.788	–	**47**.**932**	49.196
	0.5	PR	**1**.**766(0.037)**	1.828(0.037)	1.767(0.037)	1.767(0.037)
		RMSE	**0**.**150**(0.096)	1.138(**0.090**)	0.247(0.127)	0.196(0.114)
		${\text{AMSE}}_{H(t)}$	**0**.**069**	2.358	0.210	0.116
		${\text{AMSE}}_{\mu (t)}$	**16**.**520**	92.745	34.048	23.640
	0.9	PR	0.928(0.032)	–	**0**.**907(0.032)**	0.913(0.032)
		RMSE	0.946(**0.070**)	–	**0**.**562**(0.162)	0.608(0.160)
		${\text{AMSE}}_{H(t)}$	2.704	–	**0**.**332**	0.628
		${\text{AMSE}}_{\mu (t)}$	157.932	–	**60**.**054**	64.797

Note: The best indicator among candidates are presented in bold. RMSE: root mean squared error; LQR: linear quantile regression; NAQR: nonprametric additive quantile regression; MLQR: misspecified linear quantile model

### 3.2. Performance of EAW-based predictors

To check the efficiency of (10), we design a series of simulations for the similar mixed effect model in Experiment 2 but with different distributions of $u_{i}$ :

Case I
$(u_{1,i},u_{2,i}{)}^{⊤}$ follows a bivariate normal distribution with the same covariance in Experiment 2;
Case II
$u_{1,i}$ and $u_{2,i}$ are independently distributed with the symmetric Laplace distribution with $\left(\sigma_{1},\sigma_{2})=(1,2\right)$ .

**Figure 4.**
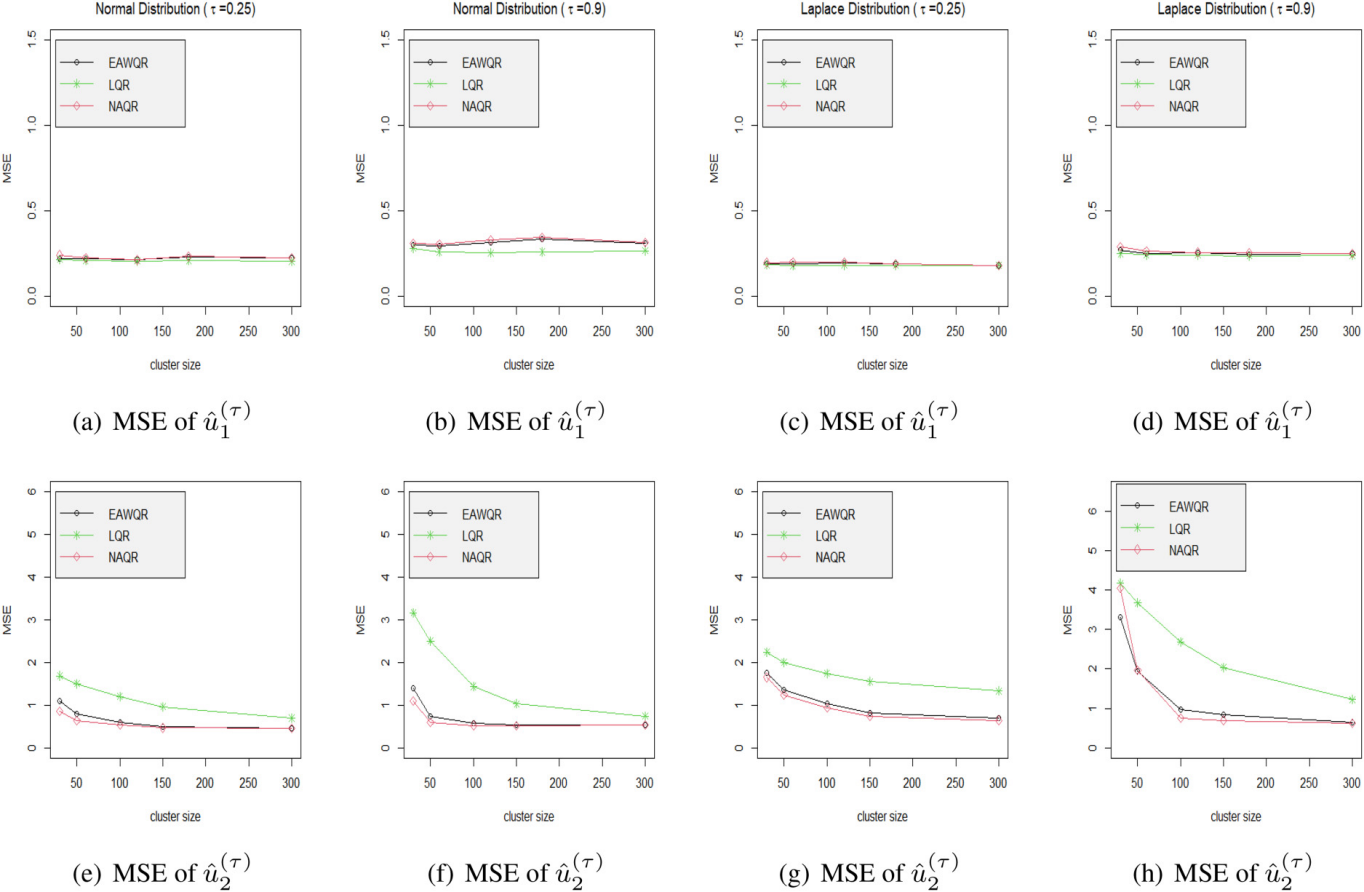
Mean squared errors of predicted random effects under different model specifications in Experiment 2. Different columns contain different distributions via the probability level be either 0.25 or 0.9.

Taking normal error as an example, the simulation is carried out in view of τ=0.25,0.5,0.9 . Other options such as candidate procedures, splitting strategies are set same as that in Experiment 2. To reflect the predicting accuracy, Table 4 reports the comparison of MSE between EAW-based BLP ${\hat{u}}^{\left(\tau \right)}$ and other separate specifications. It implies that the EAW predictor performs generally well in all probability levels, and is close to that of “correct estimating procedure” as n increases. The scatter-curves of MSEs with different cluster sizes are plotted in Figure 4, which shows that the EAW-based BLP is pretty efficient for the part of significant difference among different specifications. It is relevant to point out that when the cluster size is small, large variations of ${\hat{u}}^{\left(\tau \right)}$ ’s MSE may be enacted and we recommend to increase the number of random splits for EAW to eliminate the volatility.

**Table 4.** MSEs of BLP ${\hat{u}}^{\left(\tau \right)}$ with different distributions. Each pair (a,b) represents the MSE of $\left({\hat{u}}_{1}^{\left(\tau \right)},{\hat{u}}_{2}^{\left(\tau \right)}\right)$ respectively.

			Procedure			
n	τ	MSE	LQR	MLQR	NAQR	EAW-Prediction
120	0.25	Case I	(**0.205**, 1.198)	–	(0.211, **0.504**)	(0.211, 0.598)
		Case II	(**0.180**, 1.740)	–	(0.200, **0.931**)	(0.193, 1.024)
	0.5	Case I	(**0.186**, **0.343**)	(0.321, 0.441)	(0.195, 0.404)	(0.188, 0.350)
		Case II	(**0.163**, **0.444**)	(0.242, 0.732)	(0.164, 0.633)	(0.163, 0.469)
	0.9	Case I	(**0.253**, 1.432)	–	(0.330, **0.523**)	(0.311, 0.576)
		Case II	(0.235, 2.531)	–	(**0.231**, **0.772**)	(0.230, 0.957)
300	0.25	Case I	(**0.203**, 0.694)	–	(0.224, **0.452**)	(0.224, 0.456)
		Case II	(0.180, 1.332)	–	(**0.177**, **0.643**)	(0.177, 0.687)
	0.5	Case I	(**0.184**, **0.310**)	(0.297, 0.403)	(0.186, 0.345)	(0.185, 0.321)
		Case II	(**0.164**, **0.403**)	(0.218, 0.537)	(0.164, 0.550)	(0.164, 0.412)
	0.9	Case I	(**0.265**, 0.740)	–	(0.312, **0.530**)	(0.308, 0.536)
		Case II	(**0.237**, 1.226)	–	(0.247, **0.620**)	(0.246, 0.639)

Note: The best indicator among candidates are presented in bold. LQR: linear quantile regression; NAQR: nonprametric additive quantile regression; MLQR: misspecified linear quantile model; MSE: mean squared error; BLP: best linear prediction.

## 4. MADIT data analysis

To illustrate the practical superiority of the EAW estimation, we apply the methodology to analyze the MADIT data. The dataset has been studies by Deng^27^ and Liu et al.^8^ Note that most of the statistical modeling and estimating are based on the particular specification, and have not explicated the outperformance of the fitted model or estimating methodologies. We will demonstrate the rationality and the advantage of our method.

The MADIT was designed to evaluate the effectiveness of an implantable cardiac defibrillator (ICD) in preventing sudden death in high-risk patients. In the collected database, a total of 181 patients from 36 centers in the United States were recruited to be fully sequential and randomly observed. Of which 89 patients were assigned to the ICD group and others were assigned to the conventional therapy group, and all of the observations were censored in large degree. Concretely, the data set consists of patient ID code, Treatment code (1 for ICD and 0 for conventional), observed survival time in days, death indicator (1 for death and 0 for censored), cost type, and daily cost from the start to the completion of the trial. Following types of cost were present in the experiment: Type 1 is for hospitalization and emergency department visits; Type 2 is for outpatient tests and procedures; Type 3 is for physician/specialist visits; Type 4 is for community services; Type 5 is for medical supplies, and Type 6 is for medications.

It is widely accepted that the medical costs are not affected by Treatment Types as they often appear in the survival part to influence the risk rate of a subject, thus we will not consider it in the outcome model. On the other hand, patients may have more than one cost types simultaneously even at the same time point, seven significant covariates $x_{1,i}(t),\ldots ,x_{7,i}(t)$ are contained, where $x_{r,i}(t)=1$ if the observation of Type r ( r=1,…,6 ) is 1 and $x_{r,i}(t)=0$ otherwise, and $x_{7,i}(t)=f(t)$ is imposed to depict the affect of the treatment time. To contrast with Liu et al.,^8^ the same data preprocessing method is adopted to make the comparison, that is, compresses the data set by summing the daily cost in terms of each 12-day periods. We set up both longitudinal quantile regression (denoted as “Liu et al.”) and additive mixed effect models (denoted as “AQMM”) to fit the MADIT data:

(16) $$\begin{array}{rl} Q_{\tau }^{\left(1\right)}(y_{i}(t)|x_{i}(t)) & =\beta_{0,\tau }+\sum_{p=1}^{6}\beta_{p,\tau }x_{p,i}(t)+\beta_{7,\tau }f(t)\\ Q_{\tau }^{\left(2\right)}(y_{i}(t)|x_{i}(t),u_{i}) & =\beta_{0,\tau }+\sum_{p=1}^{6}\beta_{p,\tau }x_{p,i}(t)+\beta_{7,\tau }f(t)+tu_{1,i}+u_{2,i} \end{array}$$

where f(t) is specified as either f(t)=t or f(t)=log(t) such that $Q_{\tau }^{\left(1\right)}(y_{i}(t)|x_{i}(t))$ represents the conditional quantile in Liu et al.^8^ In the second model $u_{i}=(u_{1,i},u_{2,i}{)}^{⊤}$ are assumed as the zero-mean random effects with a symmetric positive-definite variance-covariance matrix.

We implement EAW estimator among $Q_{\tau }^{\left(1\right)}(y_{i}(t)|x_{i}(t))$ and $Q_{\tau }^{\left(2\right)}(y_{i}(t)|x_{i}(t),u_{i})$ in (16) with 50 independent splits (the smoothing parameters h =1 and a(h)=0.01 ). Table 5 lists the estimated 5-year and total treatment period cumulative quantile costs under τ=0.25,0.5 and 0.75 , and explains similar features with those in Liu et al.^8^ Note that the fitted residuals (marked by “Res” in Table 5) illustrate that additive quantile mixed effect model (AQMM) is more suitable as it contributes the between-subject variability by means of random effects, the change of cumulative costs is relatively flat among various quantile levels. On the other hand, EAW estimator based on either Liu et al. or AQMM could maintain the optimistic result among different probability levels, while candidate models performs differently. It is consistent with the conclusions of simulation studies.

**Table 5.** Fitting residuals, 5-year and total period estimated cumulative quantile costs for complete MADIT dataset with different specifications of f(t) . Each pair (a,b) represents the estimators of Liu et al. and the AQMM, respectively.

τ	f(t)	Res	Year 1	Year 2	Year 3	Year 4	Year 5	Total
0.25	t	(0.244, 0.165)	(14994.9, 25965.9)	(22126.3, 35858.4)	(30010.7, 46002.8)	(38714.6, 55162.6)	(50143.9, 67284.4)	(50477.0, 67502.7)
	log(t)	(0.239, 0.156)	(17054.9, 25385.7)	(23851.2, 33152.1)	(31236.2, 41556.1)	(39218.9, 49856.2)	(49807.8, 61978.3)	(50122.1, 62233.1)
	EAW	(0.240, 0.163)	(15808.7, 24421.2)	(22403.7, 32936.1)	(29596.9, 41872.7)	(37403.5, 50268.9)	(47723.5, 61847.4)	(48034.7, 62075.8)
0.5	t	(0.295, 0.179)	(34057.1, 32324.2)	(49548.2, 47122.3)	(67216.7, 64547.2)	(87270.7, 82876.0)	(115124, 110822)	(115659, 111360)
	log(t)	(0.291, 0.170)	(35683.4, 34328.1)	(49955.8, 48064.4)	(65798.0, 63780.7)	(83348.8, 79930.6)	(107456, 104107)	(107954, 104611)
	EAW	(0.293, 0.173)	(33559.3, 32111.3)	(47427.9, 45482.5)	(62916.5, 60892.3)	(80154.9, 76831.5)	(103880, 100760)	(104372, 101260)
0.75	t	(0.224, 0.169)	(75748.2, 38218.7)	(109885, 55962.3)	(152986, 76972.3)	(191616, 99362.4)	(263317, 133616)	(264146, 134270)
	log(t)	(0.224, 0.164)	(76145.9, 44585.7)	(107538, 62092.7)	(146112, 82036.1)	(180552, 102626)	(242617, 133366)	(243389, 133970)
	EAW	(0.224, 0.164)	(71899.8, 39855.1)	(102233, 56616.5)	(139718, 75990.3)	(173305, 96243.2)	(234000, 126702)	(234763, 127308)

MADIT: multicenter automatic defibrillator implantation trial; AQMM: additive quantile mixed effect models; EAW: exponential aggregation weighting.

In order to inspect the efficiency of different models we randomly split 120 of the 181 subjects from the data set to train the specified models, and use the remaining 61 subjects to the out-of-sample test. The prediction test prediction risk (PT-PR) and the prediction test MSE (PT-RMSE) of the estimated $Q_{\tau }$ are proposed to test the goodness of fit for the models of MADIT data, where

$$\begin{array}{r} \text{PT-PR}(\tau )=\frac{1}{61}\sum_{i=1}^{61}\frac{1}{n_{i}}\sum_{t=1}^{n_{i}}\rho_{\tau }\left\{y_{i}(t)-{\hat{q}}_{\tau }(x_{i}(t))-t{\hat{u}}_{1,i}-{\hat{u}}_{2,i}\right\} \end{array}$$

$$\begin{array}{r} \text{PT-RMSE}(\tau )={\left[\frac{1}{61}\sum_{i=1}^{61}\frac{1}{n_{i}}\sum_{t=1}^{n_{i}}{\left\{y_{i}(t)-{\hat{q}}_{\tau }(x_{i}(t))-t{\hat{u}}_{1,i}-{\hat{u}}_{2,i}\right\}}^{2}\right]}^{1/2} \end{array}$$

$\left(y_{i}(t),x_{i}(t),t)\in \{\text{Test Dataset}\right\}$ , ${\hat{u}}_{1,i}$ , ${\hat{u}}_{2,i}$ are the respective BLP of random effects for test data and $t{\hat{u}}_{1,i}+{\hat{u}}_{2,i}=0$ for Liu et al. The split procedure is repeated by 50 independent times and the results are presented in Table 6, which visually shows that the fitting effect of different models is discrepant in different probability levels. For instance, two AQMMs report less PT-PR and PT-RMSE than those of Liu et al. in most cases, and the horizontal comparison shows that the mixed effect models with different f(t) have their own merits for different values of τ (e.g. the model with f(t)=t is better fitted as τ values far away from the median), while Liu et al. with f(t)=log(t) is more suitable than that with f(t)=t . The diversities are enlarged by importing the random effects. However, the EAW estimator overcomes the uncertainty, outperforms compared to a particular one in the case of large gap of different fitted models, the corresponding aggregated weights perform almost the same propensities as those of EAW results as well. Consequently, it can be considered as the “uniformly optimal fitting” among candidate specifications and different quantile levels for MADIT data.

**Table 6.** PT-PRs, PT-RMSEs (in parentheses) and aggregated weights (the second row of each group) for MADIT data: linear quantile models constructed in Liu et al., AQMM, and EAW-estimation.

		Specification of f(t)		
τ	Model	t	log(t)	EAW-Estimation
0.1	Liu et al.	0.137(1.523)	**0.136(1.504)**	0.136(1.510)
		0.363	**0.637**	–
	AQMM	**0.102(0.854)**	0.103(0.873)	0.102(0.862)
		**0.522**	0.478	–
0.25	Liu et al.	0.235(0.789)	**0.233(0.777)**	0.234(0.779)
		0.295	**0.705**	–
	AQMM	**0.154(0.417)**	**0.154(0.417)**	0.154(0.414)
		0.437	**0.563**	–
0.5	Liu et al.	0.279(0.568)	**0.278(0.560)**	0.278(0.562)
		0.293	**0.707**	–
	AQMM	0.168(0.269)	**0.165(0.257)**	0.166(0.260)
		0.333	**0.667**	–
0.75	Liu et al.	0.226(0.799)	**0.225(0.786)**	0.225(0.790)
		0.290	**0.710**	–
	AQMM	**0.159(0.408)**	0.160(0.440)	0.160(0.417)
		0.461	**0.539**	–
0.9	Liu et al.	0.126(1.345)	**0.126(1.333)**	0.126(1.336)
		0.423	**0.577**	–
	AQMM	**0.107(0.908)**	0.107(0.948)	0.107(0.948)
		0.460	**0.540**	–

Note: The best indicator among candidates are presented in bold. AQMM: additive quantile mixed effect models; PT-PR: prediction test prediction risk; PT-RMSE: prediction test root mean squared error; MADIT: multicenter automatic defibrillator implantation trial; EAW: exponential aggregation weighting.

## 5. Concluding remarks

This paper investigates the adaptive estimation for longitudinal quantile regression via EAW algorithm. Under time-dependent covariates and right-censored history process, EAW estimator and IPCW method are combined to derive τ -CQF for mixed effect model. Based on the secondary smoothing approximation of the check loss function, the oracle inequalities are easily established. We show that the estimator of τ -CQF is consistent as long as one of the candidate converges to the correct specification. Further, the BLP of the random effects are modified by EAW. The applicability of the method has emerged in MADIT data analysis.

It is worthy to point out that the secondary smoothing loss has some good theoretical properties: It supplies the differentiability at origin, guarantees the convexity and strong smoothness as well. On the other hand, the quadratic smooth pattern provides the analogous strong convexity as that of squared-type losses, which is essential for the risk bound under squared error loss, and simplifies the verification of the consistency of cumulative quantile function estimator. However, in practice, smoothing parameters should not be valued too large to affect the aggregated result, searching a convenient recommendation for h and a(h) among a real dataset is not an effortless work that remains to be further explored.

The idea of aggregation is not difficult to be extended into fixed effect models: when the individual effect is specified, candidates of $q_{\tau }(x(t))$ are estimated by the corresponding methods (Kato et al.^30^ and Koenker,^9^ among others) and EAW estimator is weighted from each ${\hat{q}}_{\tau }^{\left(k\right)}(x(t))$ . Due to the layout we did not discuss this in detail, which will be potential in the sense of complex censoring mechanisms as well. Through the paper we use a nonparameter estimation for censoring and the methods in Galvao et al.,^6^ Harding and Lamarche^7^ are both under a specific procedure of the mechanism. One can naturally generalize it as a multiply robust case when both the conditional quantile and the response probability tend to be misspecified. Besides, the aggregated estimator of (4) depends on the linear structure of random effects and we wonder how sensitive is the performance to varying degrees of misspecification. These will become valuable study in our further research.

## Supplemental Material

sj-zip-1-smm-10.1177_09622802231164730 - Supplemental material for Adaptive aggregation for longitudinal quantile regression based on censored history process Supplemental material, sj-zip-1-smm-10.1177_09622802231164730 for Adaptive aggregation for longitudinal quantile regression based on censored history process by Wei Xiong, Dianliang Deng, Dehui Wang and Wanying Zhang in Statistical Methods in Medical Research

## Appendix

In Appendix, we give the proof of theoretical results in main sections. As we considered the secondary approximation of $\rho_{\tau }(\cdot )$ in aggregation procedure, the following lemma presents the L -smoothness and strong convexity of $\rho_{\tau ,h}^{*}(\cdot )$ , which is essential to verify the risk bounds of EAW estimator.

Lemma 1 The secondary smoothing check loss function $\rho_{\tau ,h}^{*}(v)$ is strongly convex in R , and for $v,v_{0}\in R$ ,

(A.1)
$$\begin{array}{r} \underline{c}(v-v_{0}{)}^{2}\leq \rho_{\tau ,h}^{*}(v)-\rho_{\tau ,h}^{*}(v_{0})-\rho_{\tau ,h}^{{*}^{{}^{\prime }}}(v_{0})(v-v_{0})\leq \bar{c}(v-v_{0}{)}^{2} \end{array}$$

where $\underline{c}=a(h)$ and $\bar{c}=(h^{-1}+2a(h))/2$ .

**Proof**. **Proof**. To verify the strong convexity we prove the first inequality of (A.1). For the given h>0 and a(h)>0 , $\rho_{\tau ,h}^{*}(v)=\rho_{\tau ,h}(v)+a(h)v^{2}$ ,

$$\begin{array}{rl} & \rho_{\tau ,h}^{*}(v)-\rho_{\tau ,h}^{*}(v_{0})-\rho_{\tau ,h}^{{*}^{{}^{\prime }}}(v_{0})(v-v_{0})\\ & \;=\left\{\rho_{\tau ,h}(v)-\rho_{\tau ,h}(v_{0})-\rho_{\tau ,h}^{{}^{\prime }}(v_{0})(v-v_{0})\right\}+\left\{a(h)v^{2}-a(h)v_{0}^{2}-2a(h)v_{0}(v-v_{0})\right\}:=P_{1}+P_{2} \end{array}$$

Note that $P_{2}=a(h)(v-v_{0}{)}^{2}$ , we focus on the discussion of $P_{1}$ . In the case of $v\leq v_{0}<-(1-\tau )h$ , we have

$$\rho_{\tau ,h}(v)-\rho_{\tau ,h}(v_{0})-\rho_{\tau ,h}^{{}^{\prime }}(v_{0})(v-v_{0})=(\tau -1)(v-v_{0})-(\tau -1)(v-v_{0})=0$$

When $v\leq -(1-\tau )h\leq v_{0}\leq \tau h$ ,

$$\begin{array}{rl} \rho_{\tau ,h}(v)-\rho_{\tau ,h}(v_{0})-\rho_{\tau ,h}^{{}^{\prime }}(v_{0})(v-v_{0}) & =\frac{2h(\tau -1)v-h^{2}(1-\tau )-2v_{0}v+v_{0}^{2}}{2h}\\ & \geq \left\{ \begin{array}{rl} & (1-\tau {)}^{2},\;0\leq v_{0}\leq \tau h,\\ & 0,\;\;\;-(1-\tau )h\leq v_{0}\leq 0 \end{array}\right. \end{array}$$

If v≤−(1−τ)h and $v_{0}>\tau h$ ,

$$\rho_{\tau ,h}(v)-\rho_{\tau ,h}(v_{0})-\rho_{\tau ,h}^{{}^{\prime }}(v_{0})(v-v_{0})=-v-\frac{h}{2}(1-\tau {)}^{2}+\frac{h}{2}\tau^{2}\geq \frac{h}{2}$$

If $-(1-\tau )h\leq v\leq v_{0}\leq \tau h$ ,

$$\begin{array}{rl} & \;\rho_{\tau ,h}(v)-\rho_{\tau ,h}(v_{0})-\rho_{\tau ,h}^{{}^{\prime }}(v_{0})(v-v_{0})=\frac{(v-v_{0}{)}^{2}}{2h} \end{array}$$

If $-(1-\tau )h\leq v\leq \tau h\leq v_{0}$ ,

$$\rho_{\tau ,h}(v)-\rho_{\tau ,h}(v_{0})-\rho_{\tau ,h}^{{}^{\prime }}(v_{0})(v-v_{0})=\frac{(v-h\tau {)}^{2}}{2h}\geq 0$$

If $\tau h\leq v\leq v_{0}$ ,

$$\rho_{\tau ,h}(v)-\rho_{\tau ,h}(v_{0})-\rho_{\tau ,h}^{{}^{\prime }}(v_{0})(v-v_{0})=0$$

Based on the advantages we have $P_{1}\geq 0$ , and the same discussion is available for the terms of $v_{0}<v$ . Therefore, let $\underline{c}=a(h)$ , the first inequality hence be established. To prove the L -smoothness of $\rho_{\tau ,h}^{*}(v)$ , one can see that

$$\begin{array}{r} |\rho_{\tau ,h}^{{*}^{{}^{\prime }}}(v)-\rho_{\tau ,h}^{{*}^{{}^{\prime }}}(v_{0})|\leq |\rho_{\tau ,h}^{{}^{\prime }}(v)-\rho_{\tau ,h}^{{}^{\prime }}(v_{0})|+2a(h)|v-v_{0}|\leq (h^{-1}+2a(h))|v-v_{0}| \end{array}$$

by Proposition 2 of Mkhadri et al.^31^ Therefore, the inequalities of the lemma holds by combining Definition 2.1.2 and Theorem 2.1.5 of Nesterov.^32^

**Proof of Theorem 1**. **Proof of Theorem 1**. For the aggregated estimator under a particular split process (denotes by b -split), let

$$p_{n_{0}}^{n}=\sum_{\delta_{k}\in \Delta }\pi_{j}\exp\;\left\{-\lambda \sum_{i=N_{0}+1}^{n}\sum_{j=1}^{n_{i}}{\hat{L}}_{\tau ,ij}^{\left(k\right)}\right\}$$

where the footnote b in above presentation is omitted for the sake of simplicity. Observe that

$$\begin{array}{rl} p_{n_{0}}^{n} & =\sum_{\delta_{k}\in \Delta }\pi_{j}\exp\;\left\{-\lambda \sum_{j=1}^{n_{N_{0}+1}}{\hat{L}}_{\tau ,N_{0}+1,j}^{\left(k\right)}\right\}\times \frac{\sum_{\delta_{k}\in \Delta }\pi_{j}\exp\;\left\{-\lambda \sum_{i=N_{0}+1}^{N_{0}+2}\sum_{j=1}^{n_{i}}{\hat{L}}_{\tau ,ij}^{\left(k\right)}\right\}}{\sum_{\delta_{k}\in \Delta }\pi_{j}\exp\;\left\{-\lambda \sum_{j=1}^{n_{N_{0}+1}}{\hat{L}}_{\tau ,N_{0}+1,j}^{\left(k\right)}\right\}}\\ & \;\times \cdots \times \frac{\sum_{\delta_{k}\in \Delta }\pi_{j}\exp\;\left\{-\lambda \sum_{i=N_{0}+1}^{n}\sum_{j=1}^{n_{i}}{\hat{L}}_{\tau ,ij}^{\left(k\right)}\right\}}{\sum_{\delta_{k}\in \Delta }\pi_{j}\exp\;\left\{-\lambda \sum_{i=N_{0}+1}^{n-1}\sum_{j=1}^{n_{i}}{\hat{L}}_{\tau ,ij}^{\left(k\right)}\right\}}=\prod_{i=N_{0}+1}^{n}\left[\sum_{\delta_{k}\in \Delta }{\hat{\Omega }}_{k,i}^{b}\exp\;\left\{-\lambda \sum_{j=1}^{n_{i}}{\hat{L}}_{\tau ,ij}^{\left(k\right)}\right\}\right] \end{array}$$

For the fixed individual $i\in \{N_{0}+1,\ldots ,n\}$ , regard K as the discrete random variable and ξ as the respective discrete measure on $Z^{+}$ such that $\xi (k)=\mathrm{Pr}(K=k)={\hat{\Omega }}_{k,i}^{b}$ , k≥1 . We have

$$\sum_{\delta_{k}\in \Delta }{\hat{\Omega }}_{k,i}^{b}\exp\;\left\{-\lambda \sum_{j=1}^{n_{i}}{\hat{L}}_{\tau ,ij}^{\left(K\right)}\right\}=E_{\xi }\exp\;\{\lambda h^{*}(K)\}$$

where $h^{*}(K)=-\sum_{j=1}^{n_{i}}\rho_{\tau ,h}^{*}(y_{i}(t_{ij})-{\hat{q}}_{\tau ,b}^{\left(K\right)}(x_{i}(t_{ij})))$ . Denote $h(K)=-\sum_{j=1}^{n_{i}}\rho_{\tau }(y_{i}(t_{ij})-{\hat{q}}_{\tau ,b}^{\left(K\right)}(x_{i}(t_{ij})))$ , it can be shown that

$$\begin{array}{rl} h^{*}(K) & \leq h(K)+\frac{hT}{2}\max\{\tau^{2},(1-\tau {)}^{2}\}-a(h)\sum_{j=1}^{n_{i}}{\left\{y_{i}(t_{ij})-{\hat{q}}_{\tau ,b}^{\left(K\right)}(x_{i}(t_{ij}))\right\}}^{2}\\ & \leq h(K)+\frac{hT}{2}\max\{\tau^{2},(1-\tau {)}^{2}\} \end{array}$$

by Proposition 1 of Mkhadri et al.^31^ On the other hand, let $\mu_{k}(X)$ be the k th central moment of X ( k≥2 ), Lemma 3.6.1 of Catoni^33^ indicates

$$\begin{array}{rl} \log\;\left(E_{\xi }\exp\;\{\lambda h^{*}(K)\}\right) & \leq \lambda E_{\xi }h(K)+\frac{\lambda^{2}}{2}\mu_{2,\xi }\{h^{*}(K)\}\\ & \;\cdot \exp\;\left[\lambda \cdot \max\left\{0,\underset{\gamma \in [0,\lambda ]}{\sup}\frac{\mu_{3,\xi_{\gamma }}\{h^{*}(K)\}}{\mu_{2,\xi_{\gamma }}\{h^{*}(K)\}}\right\}\right]+\frac{\lambda hT}{2}\max\{\tau^{2},(1-\tau {)}^{2}\} \end{array}$$

with the induced measure $\xi_{\gamma }$ ( γ∈[0,λ] ) given by

$$\xi_{\gamma }(k)=\frac{{\hat{\Omega }}_{k,i}^{b}\exp\;\{\gamma h^{*}(k)\}}{\sum_{\delta_{k}\in \Delta }{\hat{\Omega }}_{k,i}^{b}\exp\;\{\gamma h^{*}(k)\}},\;\text{for }j\geq 1$$

Using Lemma 1, we have

$$\begin{array}{rl} \underset{\gamma \in [0,l]}{\sup}\frac{\mu_{3,\xi_{\gamma }}\{h^{*}(K)\}}{\mu_{2,\xi_{\gamma }}\{h^{*}(K)\}} & \leq \underset{k_{1},k_{2}\geq 1}{\sup}\left|h^{*}(k_{1})-h^{*}(k_{2})\right|\\ & \leq 2\underset{k\geq 1}{\sup}\sum_{j=1}^{n_{i}}\left|L_{\tau ,ij}^{{}^{\prime }}\right|\cdot \left|{\hat{q}}_{\tau ,b}^{\left(k\right)}(x_{i}(t_{ij}))-q_{\tau }(x_{i}(t_{ij}))\right|+2\bar{c}\underset{k\geq 1}{\sup}\sum_{j=1}^{n_{i}}{\left|{\hat{q}}_{\tau ,b}^{\left(k\right)}(x_{i}(t_{ij}))-q_{\tau }(x_{i}(t_{ij}))\right|}^{2} \end{array}$$

where $L_{\tau ,ij}^{{}^{\prime }}=\rho_{\tau ,h}^{{*}^{{}^{\prime }}}(y_{i}(t_{ij})-q_{\tau }(x_{i}(t_{ij})))$ , On the other hand,

$$\begin{array}{rl} \mu_{2,\xi }\{h^{*}(K)\} & \leq \sum_{j=1}^{n_{i}}E_{\xi }{\left[{\hat{L}}_{\tau ,ij}^{\left(K\right)}-\rho_{\tau ,h}^{*}\left(y_{i}(t_{ij})-E_{\xi }{\hat{q}}_{\tau }^{\left(K\right)}(x_{i}(t_{ij}))\right)\right]}^{2}\\ & \leq \sum_{j=1}^{n_{i}}{\left\{\left|L_{\tau ,ij}^{{}^{\prime }}\right|+4\bar{c}\underset{k\geq 1}{\sup}\left|{\hat{q}}_{\tau ,b}^{\left(k\right)}(x_{i}(t_{ij}))-q_{\tau }(x_{i}(t_{ij}))\right|\right\}}^{2}\cdot {\underline{c}}^{-1}\sum_{j=1}^{n_{i}}\left\{E_{\xi }{\hat{L}}_{\tau ,ij}^{\left(K\right)}-\rho_{\tau ,h}^{*}\left(y_{i}(t_{ij})-E_{\xi }{\hat{q}}_{\tau ,b}^{\left(K\right)}(x_{i}(t_{ij}))\right)\right\} \end{array}$$

we can obtain that

(A.2)
$$\begin{array}{rl} \log\;\left(E_{\xi }\exp\;\{\lambda h^{*}(K)\}\right) & \leq \frac{\lambda^{2}}{\underline{c}}\left(\sum_{j=1}^{n_{i}}|\zeta_{i}(t_{ij}){|}^{2}+16{\bar{c}}^{2}TC_{\tau }^{2}\right)\cdot \exp\;\left(2\lambda C_{\tau }\sum_{j=1}^{n_{i}}|\zeta_{i}(t_{ij})|+2\lambda \bar{c}TC_{\tau }^{2}\right)\\ & \;\cdot \sum_{j=1}^{n_{i}}\left\{E_{\xi }{\hat{L}}_{\tau ,ij}^{\left(K\right)}-\rho_{\tau ,h}^{*}\left(y_{i}(t_{ij})-E_{\xi }{\hat{q}}_{\tau ,b}^{\left(K\right)}(x_{i}(t_{ij}))\right)\right\}+\lambda E_{\xi }h(K)+\frac{\lambda hT}{2}\max\{\tau^{2},(1-\tau {)}^{2}\} \end{array}$$

with probability one, where $\zeta_{i}(t_{ij})=L_{\tau ,ij}^{{}^{\prime }}$ . Note that

$$\zeta_{i}(t_{ij})=\left\{ \begin{array}{rl} & \tau -1+2a(h)e_{i}(t_{ij}),\;\;e_{i}(t_{ij})<-(1-\tau )h\\ & (h^{-1}+2a(h))e_{i}(t_{ij}),\;\;-(1-\tau )h\leq e_{i}(t_{ij})\leq \tau h\\ & \tau +2a(h)e_{i}(t_{ij}),\;\;\;\;e_{i}(t_{ij})>\tau h \end{array}\right.$$

by taking the expectation (denoted by $E_{i}$ ) for both sides of above inequality with respect to individuals $y_{i}$ conditional on $x_{i}\cup (x_{k},y_{k}{)}_{k=1}^{i-1}$ , we have for p≥1 ,

$$\begin{array}{rl} {\left\|\sum_{j=1}^{n_{i}}|\zeta_{i}(t_{ij})|\right\|}_{\text{SEXP}} & \leq T\underset{p\geq 1}{\sup}p^{-1}{\left\{E_{i}\left(\max_{t_{ij}}|\zeta_{i}(t_{ij}){|}^{p}\right)\right\}}^{1/p}\\ & \leq T{\bar{K}}_{\zeta } \end{array}$$

by Minkowski inequality, where ${\bar{K}}_{\zeta }=\max\{(1-\tau )+2a(h)K_{\tau },\tau +2a(h)K_{\tau }\}$ . Let $\zeta_{i}^{C}=\sum_{j=1}^{n_{i}}|\zeta_{i}(t_{ij})|-E_{i}\sum_{j=1}^{n_{i}}|\zeta_{i}(t_{ij})|$ , using Lemma 5.15 and Remark 5.18 of Vershynin,^34^ for $c=(2e{)}^{-1}$ and $C=2e^{2}$ , we have

$${\left\|\zeta_{i}^{C}\right\|}_{\text{SEXP}}\leq 2{\left\|\sum_{j=1}^{n_{i}}|\zeta_{i}(t_{ij})|\right\|}_{\text{SEXP}}\leq 2T{\bar{K}}_{\zeta }$$

and for $|t|\leq c/\|\zeta_{i}^{C}{\|}_{\text{SEXP}}$ ,

$$\begin{array}{r} E_{i}\exp\;\left(t\zeta_{i}^{C}\right)\leq \exp\;\left(2t^{2}CT{\bar{K}}_{\zeta }\right) \end{array}$$

Hence

$$\begin{array}{rl} E_{i}\exp\;\left\{t\sum_{j=1}^{n_{i}}|\zeta_{i}(t_{ij})|\right\} & \leq \exp\;\left(2t^{2}CT{\bar{K}}_{\zeta }\right)\cdot \exp\;(tT{\bar{K}}_{\zeta }) \end{array}$$

On the other hand, the similar discussion of Lemma 2 of Gu and Zou^22^ can be used to prove for $2|t|\leq c/\|\zeta_{i}^{C}{\|}_{\text{SEXP}}$ ,

$$\begin{array}{r} E_{i}\left\{\sum_{j=1}^{n_{i}}|\zeta_{i}(t_{ij}){|}^{2}\exp\;\left(t\sum_{j=1}^{n_{i}}|\zeta_{i}(t_{ij})|\right)\right\}\leq \sqrt{2}(4TK_{\tau }{)}^{2}\exp\;(2t^{2}CT{\bar{K}}_{\zeta }+tT{\bar{K}}_{\zeta }) \end{array}$$

Since above inequality holds as $2\lambda C_{\tau }\leq c/(2T{\bar{K}}_{\zeta })$ , denote that $M=\exp\;(8\lambda^{2}C_{\tau }^{2}CT{\bar{K}}_{\zeta }+2\lambda C_{\tau }T{\bar{K}}_{\zeta })$ and $H_{1}=\max\{\tau^{2},(1-\tau {)}^{2}\}\cdot hT$ , the inequality (A.2) further can be written as

$$\begin{array}{r} E_{i}\log\;\left[E_{\xi }\exp\;\left\{-\lambda \sum_{j=1}^{n_{i}}\rho_{\tau }\left(y_{i}(t_{ij})-{\hat{q}}_{\tau ,b}^{\left(K\right)}(x_{i}(t_{ij}))\right)\right\}\right]\leq -\lambda E_{i}\sum_{j=1}^{n_{i}}\rho_{\tau ,h}^{*}\left(y_{i}(t_{ij})-E_{\xi }{\hat{q}}_{\tau ,b}^{\left(K\right)}(x_{i}(t_{ij}))\right)+\lambda H_{1} \end{array}$$

when ${\underline{c}}^{-1}\lambda^{2}\exp\;(2\lambda \bar{c}TC_{\tau }^{2})\{\sqrt{2}(4TK_{\tau }{)}^{2}M+16{\bar{c}}^{2}TC_{\tau }^{2}M\}\leq \lambda$ . Therefore,

$$\begin{array}{r} E\{\log\;(1/p_{n_{0}}^{n})\}\geq \lambda E\sum_{i=N_{0}+1}^{n}E_{i}\left\{\sum_{j=1}^{n_{i}}\rho_{\tau }\left(y_{i}(t_{ij})-\sum_{\delta_{k}\in \Delta }{\hat{\Omega }}_{k,i}^{b}{\hat{q}}_{\tau ,b}^{\left(K\right)}(x_{i}(t_{ij}))\right)\right\}-\frac{\lambda (n-N_{0})}{2}H_{1} \end{array}$$

In addition we have

$$\begin{array}{rl} E\log\;(1/p_{n_{0}}^{n}) & \leq \lambda (n-N_{0})E\int \rho_{\tau ,h}^{*}\left(y(t)-{\hat{q}}_{\tau }^{\left(k\right)}(x(t))\right)dN(t)+\log\;(1/\pi_{k})\\ & \leq \lambda (n-N_{0})E\int \rho_{\tau }\left(y(t)-{\hat{q}}_{\tau }^{\left(k\right)}(x(t))\right)dN(t)+\log\;(1/\pi_{k})+\frac{\lambda (n-N_{0})}{2}H_{1} \end{array}$$

for each $\delta_{k}\in \Delta$ , thus

$$\begin{array}{rl} & \frac{1}{n-N_{0}}\sum_{i=N_{0}+1}^{n}E\int \rho_{\tau }\left(y_{i}(t)-\sum_{\delta_{k}\in \Delta }{\hat{\Omega }}_{k,i}^{b}{\hat{q}}_{\tau ,b}^{\left(K\right)}(x_{i}(t))\right)dN_{i}(t)\\ & \;\leq E\int \rho_{\tau }\left(y(t)-{\hat{q}}_{\tau }^{\left(k\right)}(x(t))\right)dN(t)+\frac{\log\;(1/\pi_{k})}{\lambda (n-N_{0})}+H_{1} \end{array}$$

The above inequality is available for any b th split in Algorithm 1, and by the convexity of the check loss,

$$\begin{array}{r} \rho_{\tau }\left(y_{i}(t_{ij})-{\hat{q}}_{\tau }^{M}(x_{i}(t_{ij}))\right)\leq \frac{1}{B}\sum_{b=1}^{B}\frac{1}{n-N_{0}}\sum_{i=N_{0}+1}^{n}\rho_{\tau }\left(y_{i}(t_{ij})-\sum_{\delta_{k}\in \Delta }{\hat{\Omega }}_{k,i}^{b}{\hat{q}}_{\tau ,b}^{\left(k\right)}(x_{i}(t_{ij}))\right) \end{array}$$

hence (11) in Theorem 1 holds by a simple algebraic arrangement. To prove (12), we use the first inequality of Lemma 1 to obtain

$$\begin{array}{rl} \int \left\{\rho_{\tau ,h}^{*}\left(y(t)-{\hat{q}}_{\tau }^{M}(x(t))\right)-\rho_{\tau ,h}^{*}\left(y(t)-q_{\tau }(x(t))\right)\right\}dN(t) & \geq \int \rho_{\tau ,h}^{{*}^{{}^{\prime }}}\left(y(t)-q_{\tau }(x(t))\right)\left\{q_{\tau }(x(t))-{\hat{q}}_{\tau }^{M}(x(t))\right\}dN(t)\\ & \;+\underline{c}\int {\left\{q_{\tau }(x(t))-{\hat{q}}_{\tau }^{M}(x(t))\right\}}^{2}dN(t) \end{array}$$

Since the secondary approximated loss function has the same minimum point with that of check loss in R , $E[\int \rho_{\tau ,h}^{{*}^{{}^{\prime }}}(y(t)-q_{\tau }(x(t)))dN(t)|x]=0$ . Therefore,

$$\begin{array}{rl} & E\int \rho_{\tau ,h}^{*}\left(y(t)-{\hat{q}}_{\tau }^{M}(x(t))\right)dN(t)-E\int \rho_{\tau ,h}^{*}\left(y(t)-q_{\tau }(x(t))\right)dN(t)\\ & \;\geq \underline{c}E\int {\left\{{\hat{q}}_{\tau }^{M}(x(t))-q_{\tau }(x(t))\right\}}^{2}dN(t) \end{array}$$

and for $\delta_{k}\in \Delta$ , the analogous inference leads that

$$\begin{array}{rl} & E\int \rho_{\tau ,h}^{*}\left(y(t)-{\hat{q}}_{\tau }^{\left(k\right)}(x(t))\right)dN(t)-E\int \rho_{\tau ,h}^{*}\left(y(t)-q_{\tau }(x(t))\right)dN(t)\\ & \;\leq \bar{c}E\int {\left\{{\hat{q}}_{\tau }^{\left(k\right)}(x(t))-q_{\tau }(x(t))\right\}}^{2}dN(t) \end{array}$$

Repeating the previous proof by using $\rho_{\tau ,h}^{*}$ only, we have

$$\begin{array}{r} E\int \rho_{\tau ,h}^{*}\left(y-{\hat{q}}_{\tau }^{M}(x(t))\right)dN(t)\leq \underset{\delta_{k}\in \Delta }{\inf}\left\{E\int \rho_{\tau ,h}^{*}\left(y-{\hat{q}}_{\tau }^{\left(k\right)}(x(t))\right)dN(t)+\frac{\log\;(1/\pi_{k})}{\lambda (n-N_{0})}\right\} \end{array}$$

which directly illustrates (12). The proof is completed.

**Proof of Theorem 2**. **Proof of Theorem 2**. By the definition of τ -QSF, one can obtain that

$$\begin{array}{rl} {\hat{H}}_{\tau }(t)-H_{\tau }(t) & =\frac{1}{n}\sum_{i=1}^{n}\frac{I(T_{i}^{*}\geq t)}{\hat{K}(t)}{\hat{q}}_{F,\tau }^{M}(x_{i}(t))-H_{\tau }(t)\\ & =\frac{1}{n}\sum_{i=1}^{n}\frac{I(T_{i}^{*}\geq t)}{\hat{K}(t)K(t)}(K(t)-\hat{K}(t)){\hat{q}}_{F,\tau }^{M}(x_{i}(t))\\ & \;+\frac{1}{n}\sum_{i=1}^{n}\frac{I(T_{i}^{*}\geq t)}{K(t)}\left\{{\hat{q}}_{F,\tau }^{M}(x_{i}(t))-q_{F,\tau }(x_{i}(t))\right\}\\ & \;+\frac{1}{n}\sum_{i=1}^{n}\frac{I(T_{i}^{*}\geq t)}{K(t)}q_{F,\tau }(x_{i}(t))-H_{\tau }(t)\\ & :=I_{1}+I_{2}+I_{3} \end{array}$$

Since $q_{F,\tau }(x_{i}(t))$ is independent of the terminal time T and the censoring time C for each i , and $\left\{u_{i},i=1,\ldots ,n\right\}$ is a sequence of mean 0 random effects, $n^{-1}\sum_{i=1}^{n}q_{F,\tau }(x_{i}(t))=H_{\tau }(t)$ . Note that the inverse probability weight satisfies that $E\{I(T_{i}^{*}\geq t)/K(t)\}=1$ , the same discussion of Theorem 1 in Liu et al.^8^ exports

$$I_{3}\to 0\;\text{in probability or a.s.,}\;\text{as }n\to \infty$$

by Conditions (B.1), (B.4) and (B.5). On the other hand, Δ involves the consistent estimation for the fixed effect, without loss of generality we assume as ${\bar{q}}_{F,\tau }^{\left(1\right)}(x(t))=q_{F,\tau }(x(t))$ . By Conditions (B.3) and (B.6), one can obtain that for some fixed x(t) , $\left\|{\hat{q}}_{F,\tau }^{\left(1\right)}(x(t))-q_{F,\tau }(x(t))\|=o_{p}(1\right)$ . Hence applying Markov inequality and Holder inequality for equation (4), we have

$$\int \|{\hat{q}}_{F,\tau }^{M}(x(t))-q_{F,\tau }(x(t))\|dN(t)=o_{p}(1)$$

Thus,

$$\begin{array}{r} I_{2}\leq \frac{1}{n}\sum_{i=1}^{n}\frac{I(T_{i}^{*}\geq t)}{K_{1}(t)K_{2}(t)}\left\|{\hat{q}}_{F,\tau }^{M}(x_{i}(t))-q_{F,\tau }(x_{i}(t))\right\|=o_{p}(1) \end{array}$$

Besides,

$$\begin{array}{rl} I_{1} & \leq \frac{1}{n}\sum_{i=1}^{n}\frac{I(T_{i}^{*}\geq t)}{{\hat{K}}_{1}(t){\hat{K}}_{2}(t)K_{1}(t)K_{2}(t)}\|K(t)-\hat{K}(t)\|\\ & \;\cdot \|{\hat{q}}_{F,\tau }^{M}(x_{i}(t))\|\\ & \leq \frac{M_{0}}{a^{4}}\left\{K_{1}(t)\|K_{2}(t)-{\hat{K}}_{2}(t)\|+K_{2}(t)\|K_{1}(t)-{\hat{K}}_{1}(t)\|\right\}\\ & \to 0\;a.s \end{array}$$

when n→∞ according with Condition (B.4), Theorem 3.1 in Zeng and Cai,^29^ and Theorem 2 in Phadia and Van.^35^ Hence for any s≤T , the consistency of ${\hat{H}}_{\tau }(t)$ in the time quantum [0,s] is graduated by the Egoroff Theorem. Further, the relationship between τ -QSF and τ -CQF proclaims that ${\hat{\mu }}_{\tau }(s)$ is consistent as well, this completes the proof.
